# The XyloTron: Flexible, Open-Source, Image-Based Macroscopic Field Identification of Wood Products

**DOI:** 10.3389/fpls.2020.01015

**Published:** 2020-07-10

**Authors:** Prabu Ravindran, Blaise J. Thompson, Richard K. Soares, Alex C. Wiedenhoeft

**Affiliations:** ^1^Center for Wood Anatomy Research, USDA Forest Products Laboratory, Madison, WI, United States; ^2^Department of Botany, University of Wisconsin, Madison, WI, United States; ^3^Department of Chemistry, University of Wisconsin, Madison, WI, United States; ^4^Department of Forestry and Natural Resources, Purdue University, West Lafayette, IN, United States; ^5^Departamento de Ciências Biolôgicas (Botânica), Universidade Estadual Paulista, Botucatu, Brazil

**Keywords:** wood identification, charcoal identification, convolutional neural networks, deep learning, sustainability, forest products, computer vision

## Abstract

Forests, estimated to contain two thirds of the world’s biodiversity, face existential threats due to illegal logging and land conversion. Efforts to combat illegal logging and to support sustainable value chains are hampered by a critical lack of affordable and scalable technologies for field-level inspection of wood and wood products. To meet this need we present the XyloTron, a complete, self-contained, multi-illumination, field-deployable, open-source platform for field imaging and identification of forest products at the macroscopic scale. The XyloTron platform integrates an imaging system built with off-the-shelf components, flexible illumination options with visible and UV light sources, software for camera control, and deep learning models for identification. We demonstrate the capabilities of the XyloTron platform with example applications for automatic wood and charcoal identification using visible light and human-mediated wood identification based on ultra-violet illumination and discuss applications in field imaging, metrology, and material characterization of other substrates.

## Introduction

In 2018, global trade in forest products represented a value chain of more than 550 billion USD^1^ and was at the highest volume since record-keeping began in 1947 (Food and Agriculture Organization, 2018). This value chain includes logs, timbers, dressed lumber, veneers, finished products, comminuted wood products, pulp and pulp-derived products, wood fuel, and charcoal, among others. Illegal logging accounts for 15–30% of the global timber supply chain (Nellemann, 2012), resulting in lost revenue for source countries, governmental corruption, and unregulated degradation of forest lands. Of the illegal trade in timber, it is estimated that 80% is controlled by transnational criminal enterprises (Nellemann, 2012), making illegal logging the fourth most lucrative form of transnational crime after counterfeiting, drug trafficking, and human trafficking, and the most profitable form of transnational natural resource crime (May, 2017).

In part as a result of the global scale of illegal logging and its ties to transnational organized crime, industrial compliance with and governmental enforcement of laws and regulations governing trade in wood and wood-derived products have remained an international priority. These include the Convention on the International Trade in Endangered Species (CITES, 27 U.S.T. § 1087), the Lacey Act (18 U.S.C. § 42–43; 16 U.S.C. § 3371–3378), the European Union Timber Regulation (EUTR, No. 995/2010), Australia’s Illegal Logging Protection Act (2012), and Illegal Logging Protection Regulation (2014). There is also growing interest in “greening” the charcoal value chain (van Dam, 2017), which directly impacts the energy needs and livelihoods of one-third of the world’s population (Food and Agriculture Organization, 2019). Research and technology development in support of law enforcement and industrial compliance have emphasized predominantly laboratory-based approaches [as reviewed in (Dormontt et al., 2015; International Consortium on Combating Wildlife Crime, 2016; Schmitz et al., 2019)], but the first (and in some jurisdictions the only) step in the enforcement of provisions against illegal logging is identification or screening of products in the field, at ports, border crossings, or other points of control.

The current state-of-the-art for routine field screening of wood across the world is an entirely human enterprise using naked eye and hand lens observation of wood anatomical features (Miller et al., 2002; Koch et al., 2011; Wiedenhoeft, 2011; Yin et al., 2016;^2^ Ruffinatto and Crivellaro, 2019). Field screening of wood is severely limited by the dearth of human expertise in forensic wood analysis (Wiedenhoeft et al., 2019), and there is even less field expertise for charcoal. Affordable and scalable technologies that can either dramatically extend or obviate the need for human expertise clearly have value in solving the global field-screening limitation and effective evidence-based policy development for compliance or enforcement will require context-dependent modifications to the adopted technology.

To move away from dependence on, or to complement, human expertise, various authors have advocated for computer vision based approaches to wood and charcoal identification. Several proof-of-concept systems have been reported, relying either on laboratory-acquired images (Khalid et al., 2008; Wang et al., 2013; Filho et al., 2014; Muniz et al., 2016; Barmpoutis et al., 2018; Andrade et al., 2019), or field-acquired cell phone images (Tang et al., 2018) that are relatively variable in terms of chromatic control, total magnification, spherical aberration, and other data-quality factors reviewed in Hermanson and Wiedenhoeft (2011), with two notable forays into controlling these factors for field imaging (Hermanson et al., 2019; Andrade et al., 2020). Computer vision based wood (Ravindran et al., 2018) and charcoal identification is appealing because it is affordable (Ravindran and Wiedenhoeft, 2020) and therefore scalable, operates on an accepted source of variability in wood, its anatomy, and for wood has demonstrated potential for real-world field deployment (Ravindran et al., 2019). Realization of practical, context-specific, field deployment strategies requires a high quality computer vision platform that is affordable, flexible, and open source.

**Figure 1:**
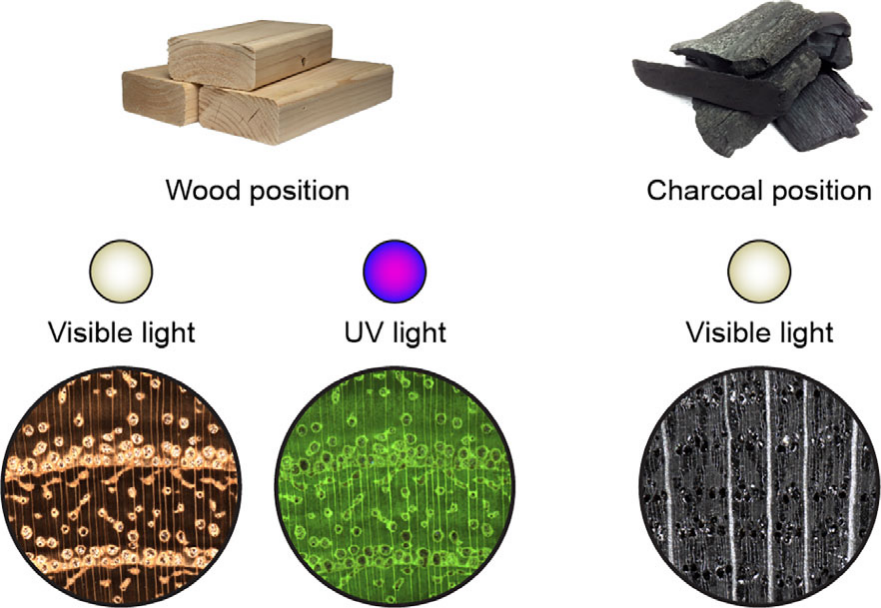
Two forest products and XyloTron macroscopic images of each, using visible light and UV light illumination. Left: 2 × 4s representing solid wood products, and visible light and UV fluorescence images of black locust (*Robinia pseudoacacia*) wood. Right: Lump charcoal with a visible light illuminated image of oak charcoal.

Here we present the XyloTron, the world’s first complete, open-source, do-it-yourself platform for imaging, identification, and metrology of materials exhibiting useful macroscopic variability, such as wood and charcoal (**Figure 1**). The XyloTron provides controlled visible light and UV illumination capability, continuously-adjustable illumination positioning, and software to control the device, capture images, and deploy trained classification models for field screening. Compared to laboratory-based methods the XyloTron exists at an affordable and scalable price point—less than 1,500 USD per unit—such that it can be deployed in the developing world (Ravindran et al., 2019), in research institutions with modest budgets, and even in classrooms. We demonstrate the capabilities of the XyloTron for two applications, namely wood identification and charcoal identification—to the best of our knowledge the first time a unified field deployable system has been used for both applications. In order to foster an ecosystem around the technology we open-source the hardware design and software applications along with the trained models at https://github.com/fpl-xylotron so the platform can be adapted to meet specific contextual needs as it is adopted.

## Materials and Methods

### XyloTron Platform

A full bill of materials, 3D design files, electrical schematics, relevant dimensioned design drawings, and a step-by-step illustrated assembly manual for building and calibrating a XyloTron system are provided in **Supplements S1** and **S2**. Each XyloTron requires a suitable laptop or desktop for image data collection and identification model deployment. Minimum hardware requirements and software necessary for data collection and field implementation are overviewed in **Supplement S3** and are available at https://github.com/fpl-xylotron.

The XyloTron has two distinct positions for its illumination array and a range of intermediate positions. When in the wood position the illumination array is as close to the specimen as possible, and when in the charcoal position, it is as distant from the specimen as possible, in this way maximizing the visibility of anatomical features for each material. The XyloTron images a fixed tissue area of 6.35 × 6.35 mm over a 2,048 × 2,048 pixel image.

### Reference Materials and Image Data Sets

#### Wood

The wood specimens in the xylarium of the USDA Forest Products Laboratory routinely serve as reference material for forensic wood identification in investigations ranging from illegal logging to arson and murder. 470 wood specimens from 31 species were selected for imaging based on the wood anatomy, surface fluorescence, and geographic origin. The transverse surfaces of the selected specimens were dry sanded to 1,500 grit then imaged using visible light with the illumination array of the XyloTron in the wood position, resulting in a dataset comprised of 3,126 non-overlapping images. In many contexts (including ours) wood identification at the species level is not possible (Gasson, 2011) and/or not required. This was leveraged to group the selected species into 12 classes for identification at a practical taxonomic granularity and to address the data scarcity problem prevalent in machine learning based wood identification.

#### Charcoal

Commercial lump charcoal specimens, submitted by the Forest Stewardship Council for forensic identification as part of a product claim verification study, were used as reference material to collect the charcoal image data set. The charcoal specimens from six genera were identified/verified by authors Soares and Wiedenhoeft using traditional methods; the selected six genera represented 74% of the European FSC-certified lump charcoal submitted. With the XyloTron illumination array in the charcoal position, 1,312 non-overlapping images of the transverse surfaces polished to 1,000 grit of 150 charcoal specimens were obtained using visible light illumination. The image dimensions and optical resolution were the same as those for the wood image data set.

### Machine Learning for Wood and Charcoal Identification

**Figure 2:**
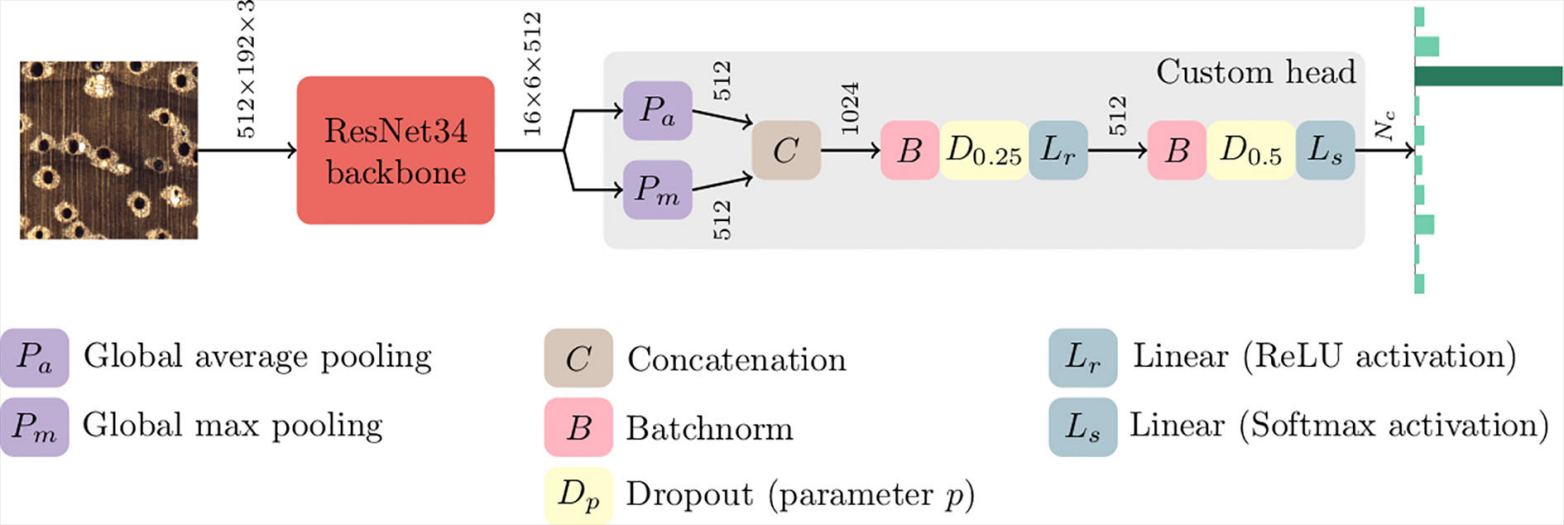
Schematic of the machine learning architecture implemented for wood and charcoal models.

Separate models [using an ImageNet (Russakovsky et al., 2015) pre-trained (He et al., 2015) backbone with custom classifier heads (see **Figure 2**)] for wood and charcoal identification were trained using a two-stage transfer learning strategy (Howard and Gugger, 2020). In the first stage, the backbone was used as a feature extractor (*i.e.*, weights frozen) and the weights of the custom head were learned, while the weights of the entire network were fine-tuned during the second stage. Both stages employed the Adam optimizer (Kingma and Ba, 2015) with simultaneous cosine annealing of the learning rate and momentum (Smith, 2018). Random image patches of size (in pixels) 2,048 × 768 were down sampled to 512 × 192 and input to the models in minibatches of size 16 with a data augmentation strategy that included horizontal/vertical flips, small rotations and cutout (Devries and Taylor, 2017). The model performance for specimen classification was evaluated using five-fold cross validation with the predicted class for a test set specimen being the majority of the class predictions for the specimen’s images. It is critical to note (as detailed in **S4**) that any given specimen contributed images only to a single fold. Further details about the species selected, their grouping into classes for wood identification, classifier architecture, training methodology, and hyperparameter settings can be found in **Supplement S4**.

## Results

### Wood Imaging Performance With Visible and UV Light

**Figure 3:**
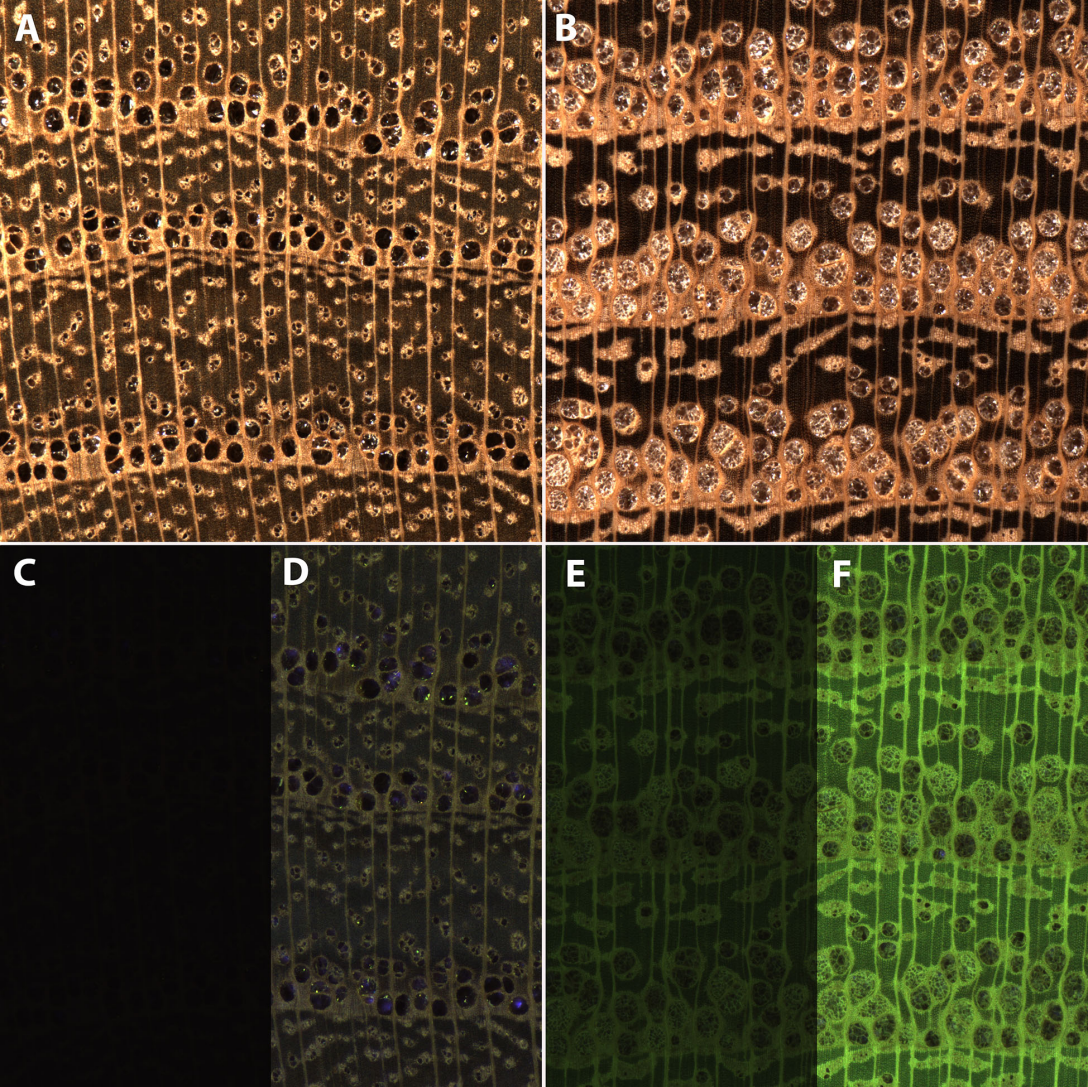
Visible light **(A, B)** and UV illumination **(C–F)** XyloTron images of *Morus rubra* **(A, C, D)** and *Robinia pseudoacacia* **(B, E, F)** with the illumination array in the wood position. **(C**–**F)** are the same tissue as above, with different camera gain and exposure times - C (0dB gain, 133 ms exposure), D (24dB gain, 133 ms exposure), E (0dB gain, 13 ms exposure), F (24dB gain, 29 ms exposure.)

*Morus rubra* and *Robinia pseudoacacia* are two species confusable at the macroscopic scale using only visible light and traditional wood anatomy (**Figures 3A, B**) but with markedly different surface fluorescence properties (**Figures 3C–F**). The visible light images of the two woods clearly depict the underlying anatomical structure. *Morus* does not exhibit surface fluorescence, so when imaged with UV illumination shows no (**Figure 3C**) or comparatively little (**Figure 3D**) anatomical detail. *Robinia*, by contrast, exhibits bright yellow-green surface fluorescence thus the images taken with UV illumination clearly show the anatomy (**Figures 3E, F**). This demonstrates the capability of the XyloTron system to image wood using visible light and to record surface fluorescence in wood substrates for identification and screening.

### Charcoal Imaging Performance With Visible Light

**Figure 4:**
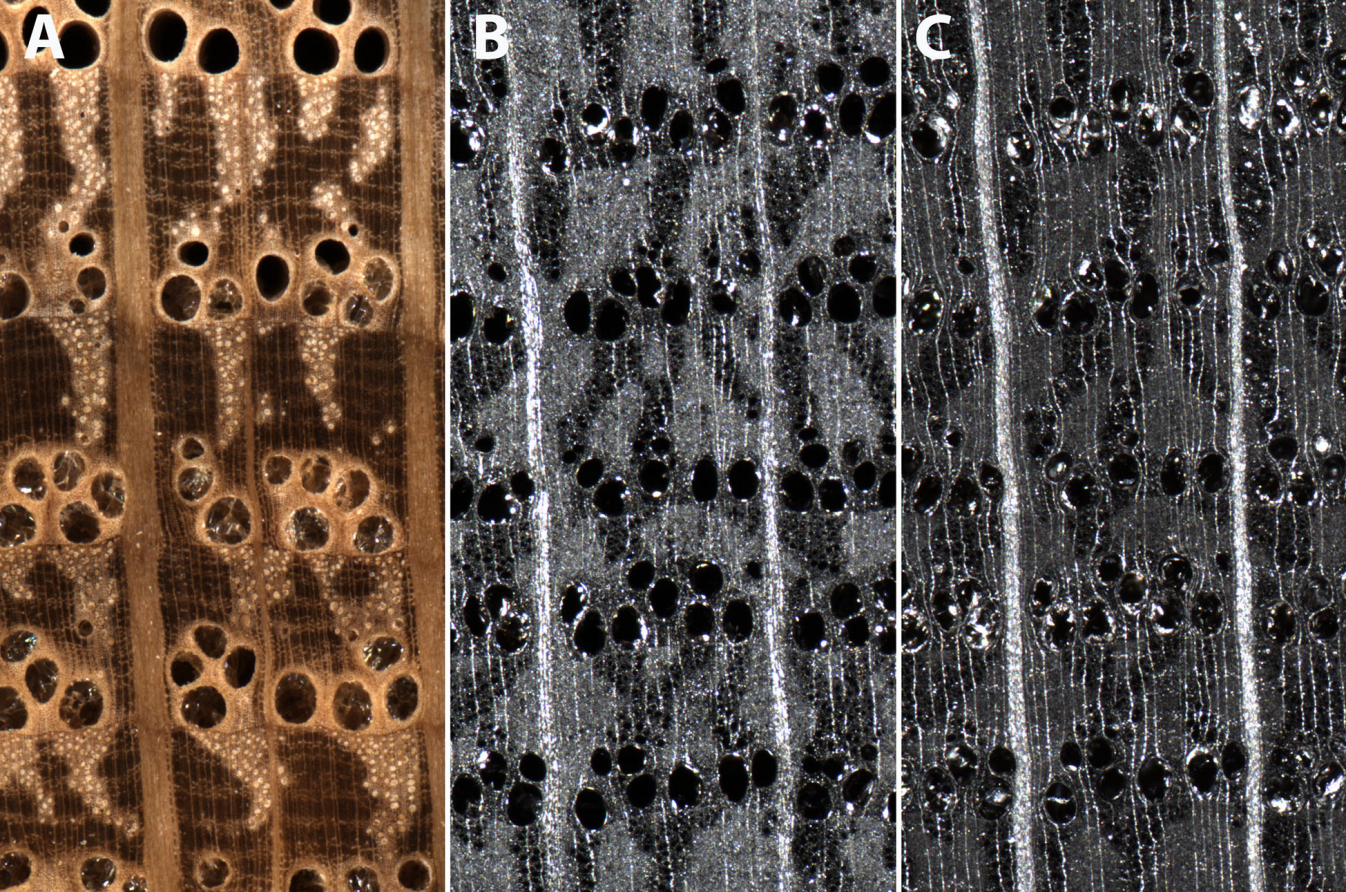
XyloTron images of *Quercus* wood **(A)** and charcoal **(B)** imaged with the illumination array in the wood position, and *Quercus* charcoal imaged with the illumination array in the charcoal position **(C)**. Fine anatomical details such as banded apotracheal parenchyma in the latewood are visible in **(A**, **C)**, but not in **(B)**.

The adjustable illumination array position of the XyloTron enables high-quality imaging of both wood and charcoal substrates. The position of the illumination array for the charcoal position was determined to provide the best visualization of wood anatomical details necessary for robust charcoal identification (**Figure 4**). The fine wood anatomical detail of the charcoal is better revealed when the illumination array is more distant from the specimen (**Figure 4C**).

### Wood and Charcoal Identification Models

**Figure 5:**
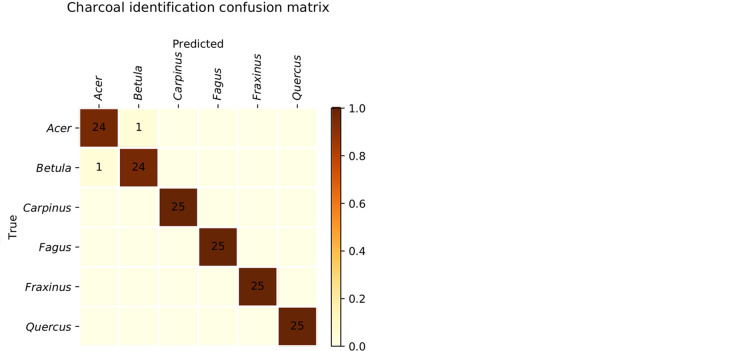
Confusion matrices for wood (left) and charcoal (right). The misclassifications in the 12 class wood model (left) mostly occur between classes with similar wood anatomy when illuminated with visible light, and all but four could be ameliorated by engaging the UV illumination capability of the XyloTron and having the human user evaluate fluorescence. The misclassifications in the 6-class charcoal identification model (right) are limited to *Acer* and *Betula*, the charcoal of which have similar anatomical features on their transverse surface.

The specimen prediction confusion matrix for the trained 12 class wood identification using the XyloTron is shown in **Figure 5** (left) with a specimen classification accuracy of 97.7%. Most of the incorrect predictions could be overcome by a human user engaging the UV illumination function and making a determination of the presence of UV fluorescence (*e.g.* confusions between *Albizia*, which is fluorescent, and *Inga*, which is not, likewise between *Robinia* and *Morus*, between *Hymenaea* and *Detarium*, and between *Ulmus rubra* and *Ulmus americana*).

The confusion matrix for the proof-of-concept 6-class charcoal identification model using visible light illumination and the illumination array in the charcoal position is shown in **Figure 5** (right). The overall accuracy of the model is 98.7%, with misclassifications limited to confusion between *Acer* and *Betula* which are macroscopically similar.

## Discussion

The wood identification model performance vastly exceeds the performance of trained field personnel and indeed approaches or exceeds expected field performance of forensic wood anatomy experts. If human XyloTron users of this model would employ UV illumination, performance would be further enhanced. In **Figure 5** (left), misclassification of only four specimens are not solved by such a human-hybrid approach employing UV illumination: *Swietenia-Detarium*, *Dialium-Ulmus americana*, *Nauclea-Tectona grandis*, and *Tectona grandis-Morus*. In other words, the effective accuracy of a human-hybrid version of our model incorporating UV illumination increases from 97.7 to 99.1%. Field accuracy at this level distinctly exceeds even the best-performing experts in the United States when performance was evaluated at the genus level (Wiedenhoeft et al., 2019).

To the best of our knowledge, there is no program anywhere in the world to train field personnel to inspect or identify charcoal despite the fact that globally the charcoal sector generates income for more than 40 million people and caters to the energy needs of more than one-third of the world’s population, predominantly in the developing world (Food and Agriculture Organization, 2019). In the absence of an existing field identification program for charcoal we cannot directly compare our model accuracy to field inspectors. By providing a highly accurate (98.7%), field-deployable, proof-of-concept model for six classes of lump charcoal that only confuses anatomically similar charcoals, we deliver the ability to inspect and verify materials that heretofore could only be assessed reliably in the laboratory or a limited group of expert wood anatomists. Ongoing work^3^ in our laboratory is addressing the breadth of charcoal taxa currently identifiable with the XyloTron, which is expected to more adequately sample the charcoals that represent the remaining 26% of the FSC-certified lump charcoal in the EU market.

**Figure 6:**
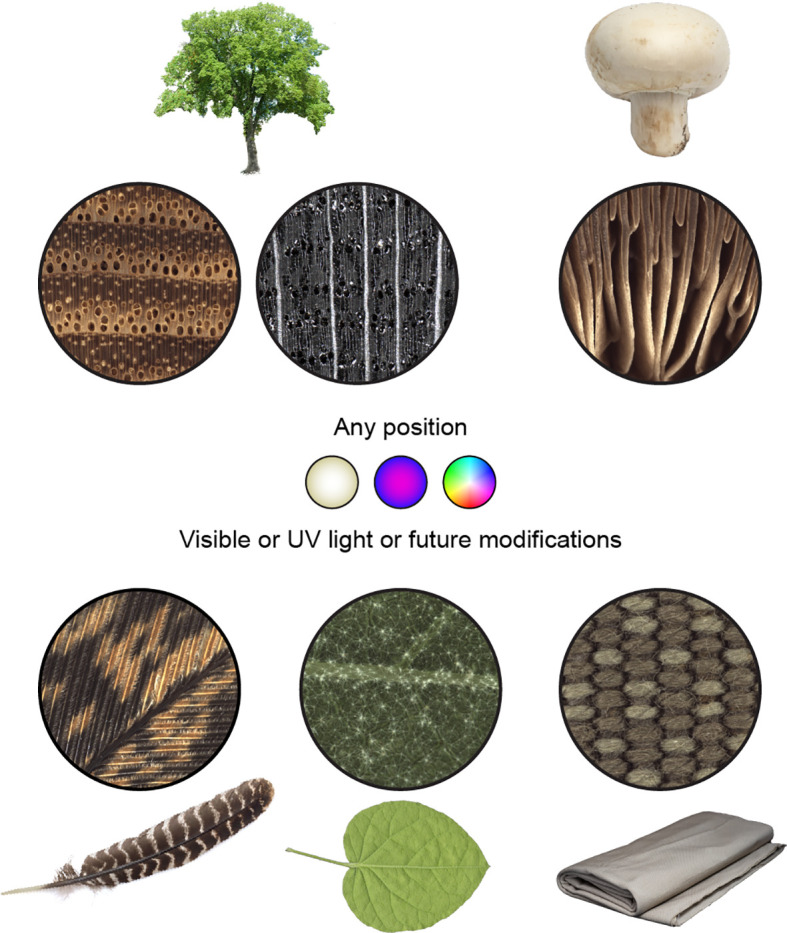
Natural and human-made materials and XyloTron macroscopic images of each, using visible light illumination with the illumination array in the wood position. In addition to wood, biological materials such as the gills on a mushroom, the rachis and barbs of a feather, and the trichomes of a leaf can be rapidly imaged to reveal macroscopic characteristics. Similarly, macroscopic features of synthetic materials, such as textiles, can be imaged for identification and characterization.

While our prior and current work with the XyloTron platform has emphasized solid wood and charcoal, we are also developing applications for wood veneers, various types of synthetic products, and field metrology. As demonstrated, the XyloTron is well-suited to capture macroscopic images of any suitable substrate with interesting macroscopic variation (**Figure 6**), and we invite researchers in a range of disciplines to adapt and enhance the platform for their applications by adding additional functionalities (e.g. multi-flash imaging, multi-color illumination, and alternative machine vision models).

## Conclusions

By providing a field-deployable system able to image and identify solid wood and charcoal, we take a step toward providing law enforcement and industrial compliance officers with the tools needed to verify wood and charcoal supply chains. Further, the open-source XyloTron platform can be readily adopted for other materials with macroscopic variability, or adapted and modified for new use-cases.

## Code Availability

The software apps for image dataset collection and trained model deployment along with the weights of the trained model will be made available at https://github.com/fpl-xylotron. The code for model training will be available from the corresponding author on reasonable request.

## Data Availability Statement

The raw data supporting the conclusions of this article will be made available by the authors on reasonable request.

## Author Contributions

PR developed the machine learning analyses. AW and BT adapted/designed/developed the XyloTron movable lighting array, PCBs, and electronics. AW and RS conducted forensic analysis of charcoal and established the scope of the identification models. PR and AW conducted data analysis, synthesis, and wrote the paper.

## Funding

This work was supported in part by a grant from the US Department of State *via* Interagency Agreement number 19318814Y0010 to AW and in part by research funding from the Forest Stewardship Council to AW. PR was partially supported by the Wisconsin Idea Baldwin Grant.

## Conflict of Interest

The authors declare that the research was conducted in the absence of any commercial or financial relationships that could be construed as a potential conflict of interest.

## Footnotes

1 http://www.fao.org/faostat/en/#data/FO

2 http://www.florestal.gov.br/servicos/85-laboratorio-de-produtos-florestais-lpf/367-madeiras-comerciais-do-brasil-chave-interativa-de-identificacao-baseada-em-caracteres-gerais-e-macroscopicos

3 See also: https://fsc.org/en/newsfeed/fsc-conducts-investigations-on-charcoal-producers.
