# Supplementary material for: The XyloTron: Flexible, Open-Source, Image-Based Macroscopic Field Identification of Wood Products
Source: Front Plant Sci. 2020 Jul 10;11:1015. doi: 10.3389/fpls.2020.01015 (PMC7366520; doi:10.3389/fpls.2020.01015)
Supplement: Supplementary file 3 [file DataSheet_3.pdf]

XyloTron Assembly and Calibration Manual

Alex C. Wiedenhoeft
Paul K. Kleinschmidt

Center for Wood Anatomy Research, Forest Products Laboratory
US Forest Service, US Department of Agriculture

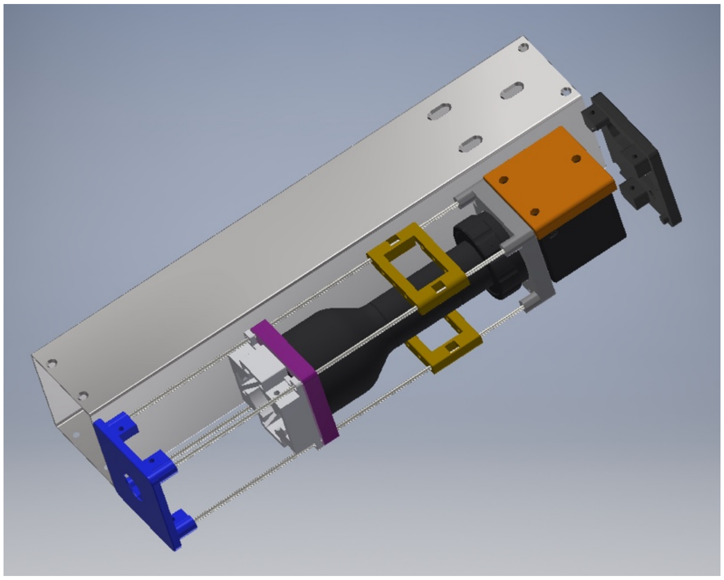

April 2020

Conventions: 3D printed part names are in **bold case**. Other distinct parts are in *italics*.
Assemblies containing one or more parts of each type are shown in ***italics and bold***.

TABLE OF CONTENTS

Part One: The Need for and Use of This Manual

Part Two: Bill of Materials

Part Three: Illustrated Parts List and Preparatory Work

Part Four: Step-by-Step XyloTron Assembly and Calibration Instructions

Part Five: Dimensioned Drawing of Machined Stainless Steel Tube

Conventions: 3D printed part names are in **bold case**. Other distinct parts are in *italics*. Assemblies containing one or more parts of each type are shown in ***italics and bold***.

PART ONE: THE NEED FOR AND USE OF THIS MANUAL

The XyloTron you are about to build is the product of several years of research, development, daily laboratory use, and field testing. Its design reflects the intent that this device can be built by an enterprising end-user to image a wide range of substrates – it is an assemblage of individually simple off-the-shelf and 3D printable components.

In Part Two we present an illustrated parts list and a short description of preparatory work that needs to be done prior to assembly. In this section we also note with an * several parts for which it is prudent to acquire more than the minimum number, because, if a mistake is made and the part is ruined, having spares facilitates the efficient completion of your XyloTron. Fortunately, those components most likely to be ruined in the learning process are among the most affordable.

The step-by-step instructions are relatively detailed. Despite this, the manual does assume certain basic skills. Specifically, no instruction on the use of basic tools (screwdrivers, files, other cutting tools) is provided, nor is there any tutorial in stripping wires or soldering electronic components. Such tutorials abound (e.g. learn.sparkfun.com). We further assume basic competence in 3D printing and that, if a printed component is faulty or damaged, such components can be readily reprinted.

This manual is intended to provide detailed instructions to assemble the XyloTron in its current incarnation. It also lays the groundwork for a novice to make special modifications themselves to adapt the XyloTron for the widest possible breadth of uses. We hope for a future where other workers develop designs superior to what we provide here, and we look forward to benefitting from those advancements in our own implementation of the XyloTron platform.

Throughout this manual, we make a distinction between 3D printed parts, the names of which will be presented in **bold** case while other parts will be presented in *italic* case. When 3D printed parts are joined with other parts, the resulting assemblies will be presented in ***bold italic*** case. This is also noted in the footer of each page. The list number of each part is presented in parentheses beside the part's name in the text.

The authors gratefully acknowledge the detailed edits and suggestions of Dr. Patricia Vega, Dr. Prabu Ravindran, and Dr. Blaise Thompson - many errors averted and improvements made. Any remaining flaws are solely the fault of the authors.

PART TWO: BILL OF MATERIALS

We present a bill of materials to build one XyloTron. Minimum possible quantity (Qty), XyloTron part name, Vendor, Manufacturer (MFR), and manufacturer part number (MFR #) are listed. Please refer to the notes in Part Three for those parts for which acquiring more than the minimum possible number might be advisable. One also needs a suitable computer, detailed in the main paper's S3.

Qty	XyloTron part	Vendor	MFR	MFR #
1	Flea 3 USB camera	FLIR	FLIR	FL3-U3-120S3C-C
1	USB 3.1 Locking Cable (Cast Metal Connectors), Type-A to Micro-B Locking Cable	FLIR	FLIR	ACC-01-2304
1	Telecentric 0.5X lens, C-mount	Edmund	Edmund Optics	63741
1	LED controller	Digikey	Recom Power	RCD-24-0.30
1	Voltage reference	Digikey	Analog Devices Inc.	AD680JTZ
1	Radial capacitor	Digikey	Murata Electronics	RDER71H104K0P1H03 B
4	White LED	Digikey	Lite-On Inc.	LTPL-P00DWS57
4	UV LED	Digikey	SunLED	XZVS54S-9A
1	SPDT switch	Digikey	NKK Switches	MS13AFG01
7	Flush mount receptacles	Digikey	Mill-Max Manufacturing Corp.	0648-0-15-15-23-27-10-0
2	Right angle JST Through-Hole 2-Pin Connector	Digikey	JST Sales America	S2B-PH-K-S(LF)(SN)
1	JST jumper two wire assembly 6"	Digikey	Sparkfun Industries	PRT-09914
4	Stainless steel rails	Grainger	Grainger	87131
1	Stainless steel tubing with inner welded seam ground smooth	McMasterCarr	McMasterCarr	89825K35
2	M3 x 0.5 x 8mm screw	McMasterCarr	McMasterCarr	92125A128
1	M3 x 0.5 x 6mm screw	McMasterCarr	McMasterCarr	92125A126
2	1/2" No. 2 screw	McMasterCarr	McMasterCarr	95893A560
7	1/4" No. 2 screw	McMasterCarr	McMasterCarr	95893A550
6	1/4" No. 0 screw	McMasterCarr	McMasterCarr	95893A505
1	Barrel Jack to 2 pin JST	Sparkfun	Sparkfun	TOL-08734
1	Barrel Jack to USB adapter cord	Sparkfun	Sparkfun	TOL-08639
1	Power distribution PCB			
1	Power input PCB			
1	Power output PCB			
4	LED PCB			

Conventions: 3D printed part names are in **bold case**. Other distinct parts are in *italics*.
Assemblies containing one or more parts of each type are shown in ***italics and bold***.

Part Three: Numbered and Illustrated Parts List and Preparatory Work

Numbered and Illustrated Parts List (number required within parentheses, * denotes prudence in acquiring more than the minimum number)

1. Front cap (1)

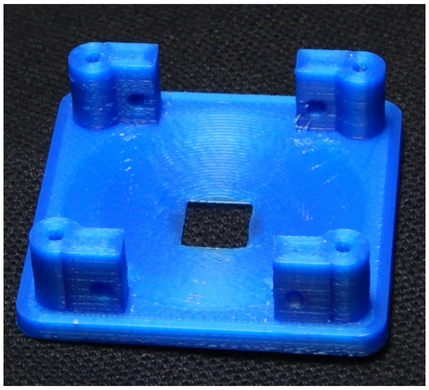

2. LED holder (1)

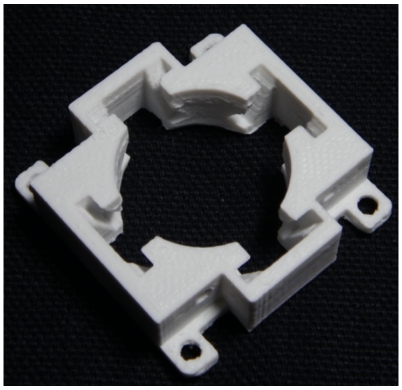

3. Lens ring (1)

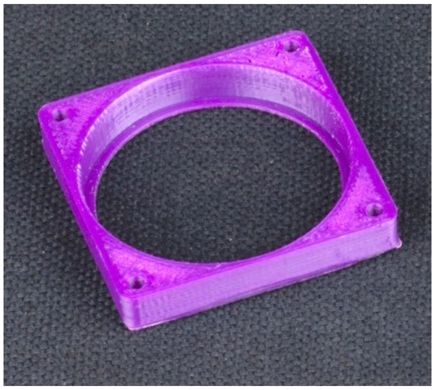

4. Sliding LED controller carriage (2)

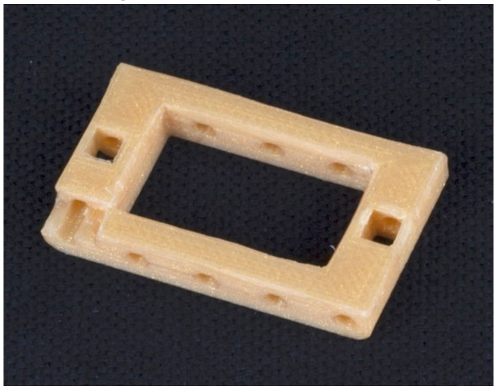

5. Flea ring (1)

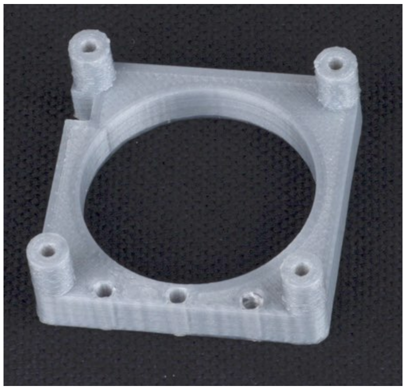

6. Camera shoe (1)

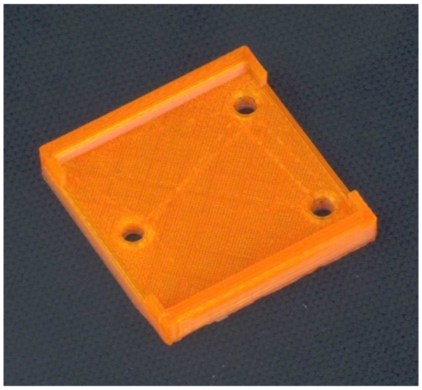

Conventions: 3D printed part names are in **bold case**. Other distinct parts are in *italics*.
Assemblies containing one or more parts of each type are shown in ***italics and bold***.

7. Back cap (1)

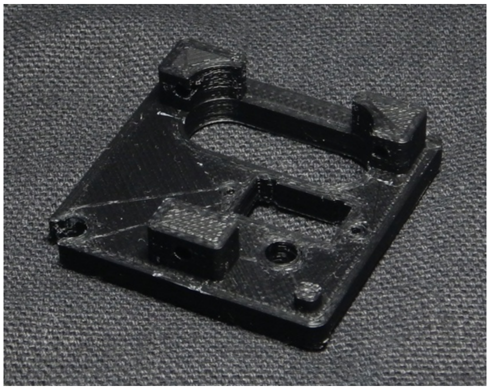

8. Flea 3 USB camera (1)

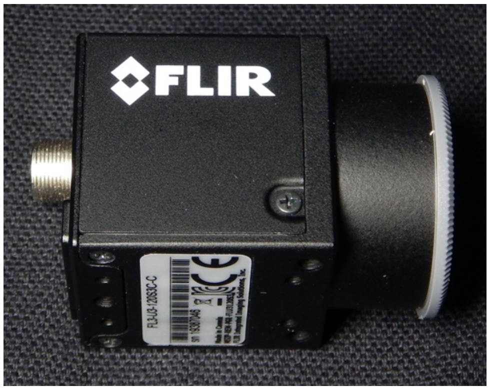

9. USB 3.1 Locking Cable (1)

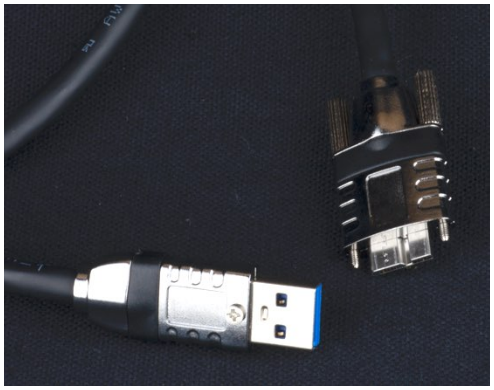

10. UV LED (4)*

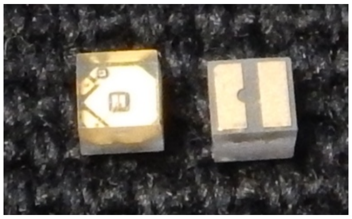

11. Telecentric 0.5X lens, C-mount (1)

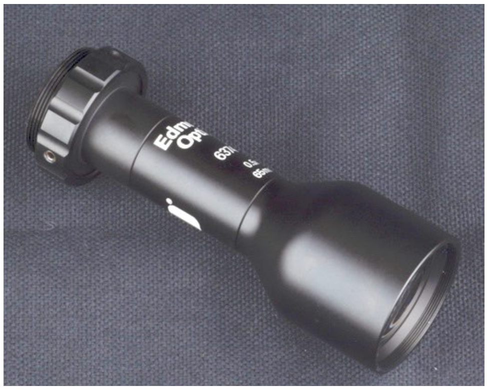

12. LED controller (1)

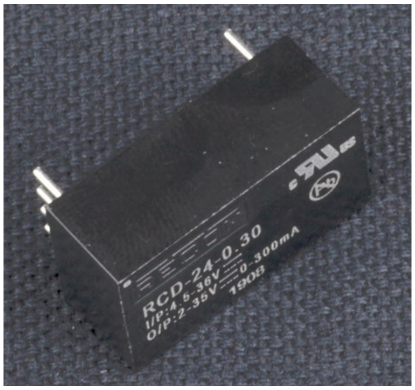

13. Radial capacitor (1)

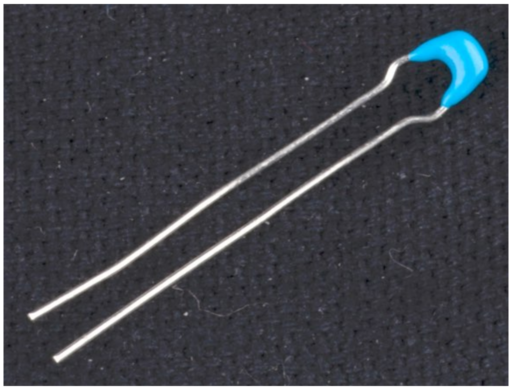

14. VIS LED (4)*

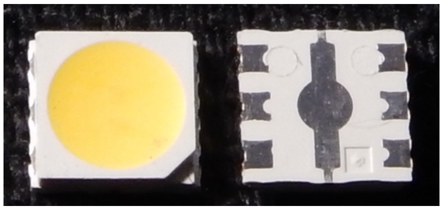

Conventions: 3D printed part names are in **bold case**. Other distinct parts are in *italics*.
Assemblies containing one or more parts of each type are shown in ***italics and bold***.

15. Switch (1)

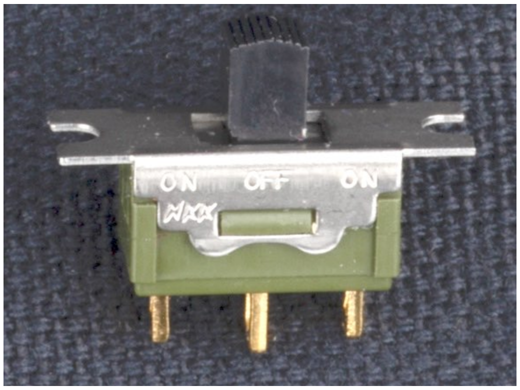

16. Right angle JST Through-Hole Connector (*)

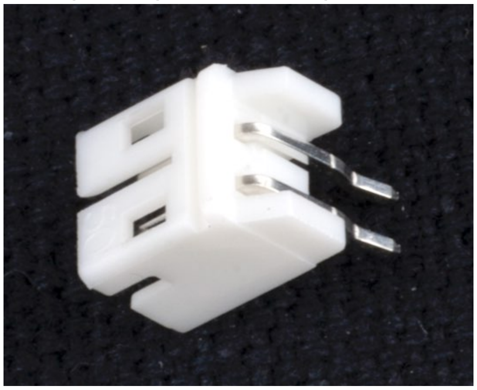

17. Stainless steel rod (4)

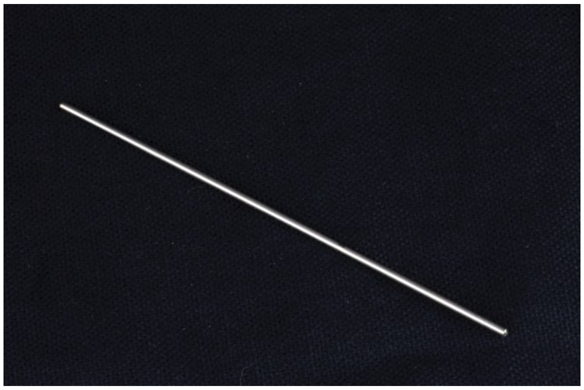

18. Machined stainless steel tube (1)

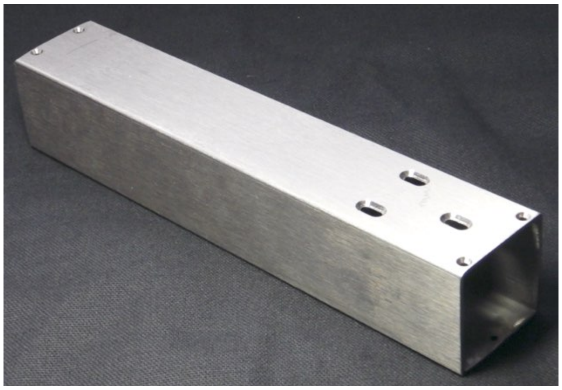

19. (a-e)
(a) 1/2″ No. 2 screw (2)
(b) 1/4″ No. 2 screw (7)
(c) 1/4″ No. 0 screw (6)
(d) 8mm camera mounting screw (2)
(e) 6mm camera mounting screw (1)

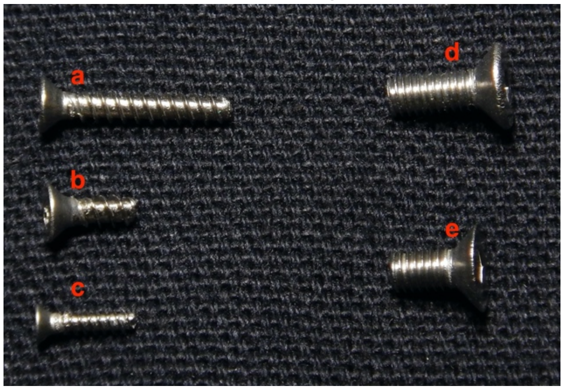

20. Voltage reference (1)

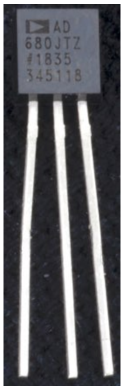

Conventions: 3D printed part names are in **bold case**. Other distinct parts are in *italics*.
Assemblies containing one or more parts of each type are shown in ***italics and bold***.

21. Press-fit receptacle (7)*

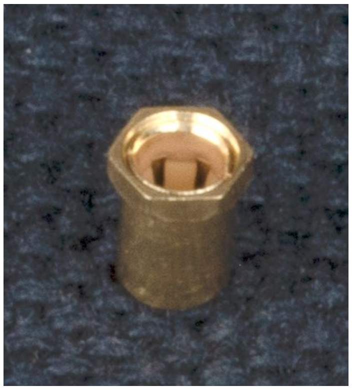

22. Barrel Jack to 2pin JST power cord (1)

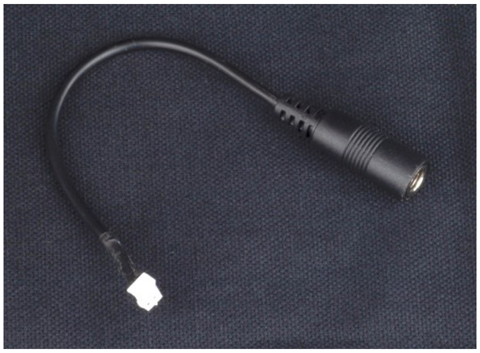

23. JST jumper two wire assembly 6" (1)

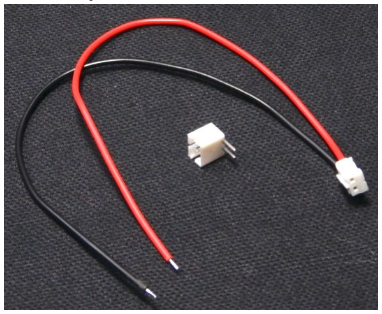

24. Barrel Jack to USB adapter cord (1)

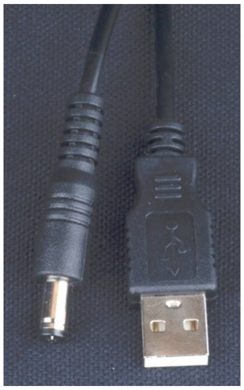

25. Power distribution PCB – top view only (1)*

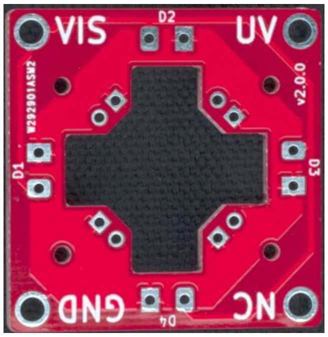

26. Power input PCB – top view, bottom view (1)*

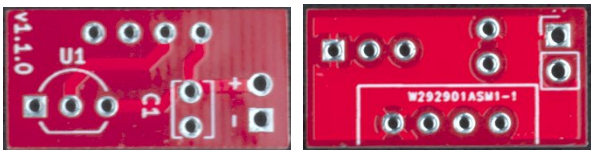

27. Power output PCB– top view, bottom view (1)*

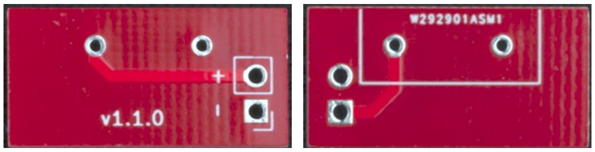

28. LED PCB (4)*

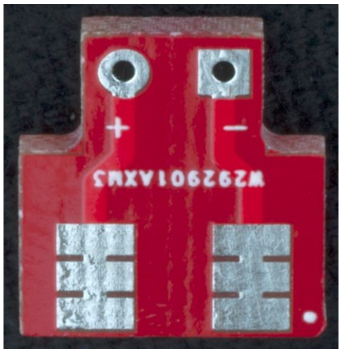

Note that for all PCBs, + through holes are round or have rounded corners, and – through holes are squares or have right-angle corners.

Conventions: 3D printed part names are in **bold case**. Other distinct parts are in *italics*.
Assemblies containing one or more parts of each type are shown in ***italics and bold***.

PREPARATORY WORK

Print the **3D printed components**. Clean them of any adhesive used in the printing process, and also do any trimming necessary - it is common that the plate face of the **3D printed component** will have a lip that is slightly wider than the rest of the part (shown below via the end view of a freshly printed **back cap (7)**). This can be removed with a utility knife or a deburring tool.

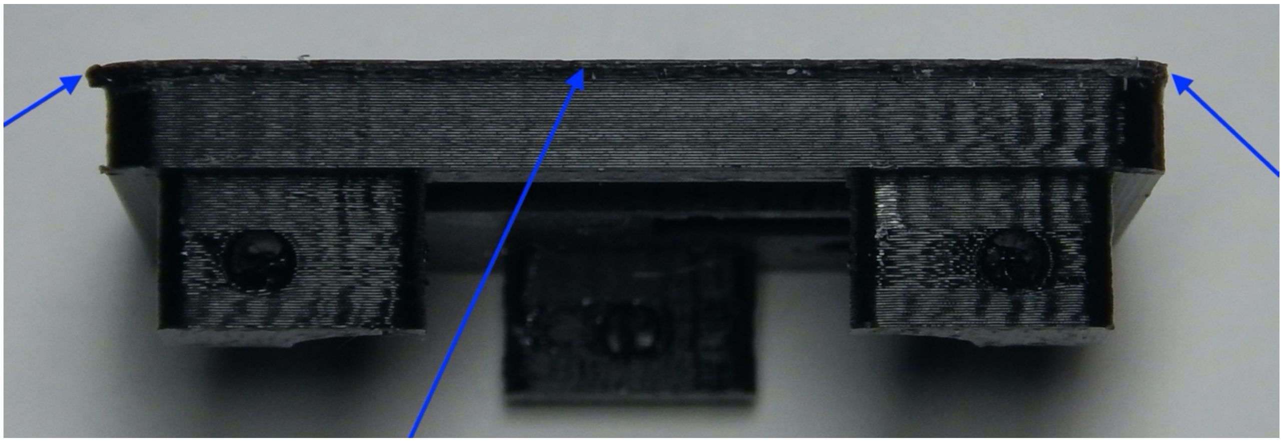

In the illustrations of this manual, each **3D printed component** is a different color. This is intended to facilitate learning the part names when comparing them to the CAD files, but it is recommended that in practice all components forward of the **lens ring** be printed in black to prevent any white-balance distortion. **3D printed components** from the **lens ring** and back can be any color that pleases the user.

Submit the Gerber files to order the *printed circuit boards*. We have printed the *boards* in red and white, but other color combinations are possible. Perhaps the most conservative selection would be black boards with dark grey printing so that the *boards* themselves cannot cause white-balance distortion.

Cut the *stainless steel rods* to length - 150-151mm – and then file or smooth the cut ends so that they are slightly rounded – this will facilitate their penetration into **3D printed parts** and *press-fit receptacles*.

Machine the *stainless steel tube*. Dimensioned drawings for the *stainless steel tube* are presented in Part Five. Ensure that the inner welded seam is on the lateral walls that require no machining and is filed or ground down smooth, and that the tube is cleaned of all metal filings, dirt, and oils.

Part Four: Step-by-Step XyloTron Assembly and Calibration Instructions

Mechanical assembly of the ***camera-lens-rods-shoe assembly***

1. Unscrew C- mount cap from the *camera* (8) throat.

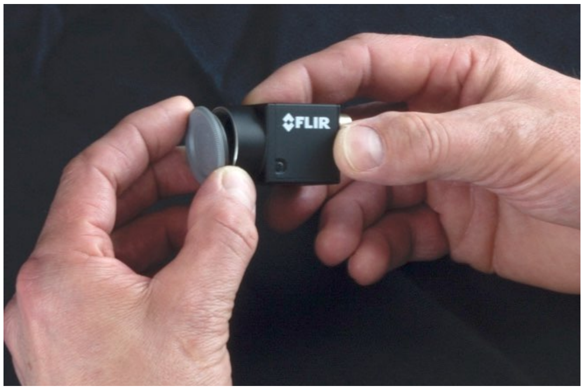

2. Slide **Flea ring** (5) over the throat of the *camera* (8) with posts facing outward in the direction of the C-mount.

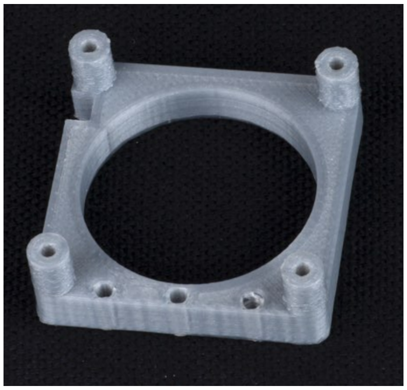

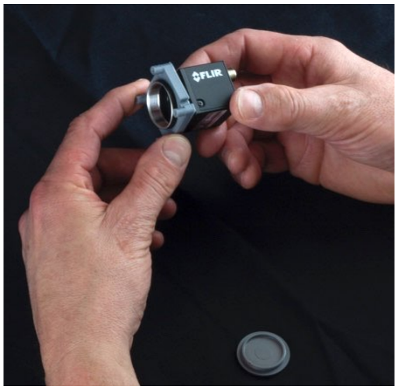

3. Unscrew C-mount cap from *lens (11)*.

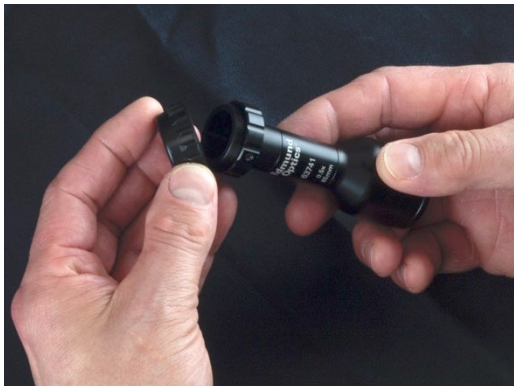

Conventions: 3D printed part names are in **bold case**. Other distinct parts are in *italics*.
Assemblies containing one or more parts of each type are shown in ***italics and bold***.

4. Thread *lens (11)* into the C-mount of the *camera* (8) and tighten snugly – the **Flea ring (5)** will now be constrained to rotate about and slide forward and backward along the *camera* (8) throat.

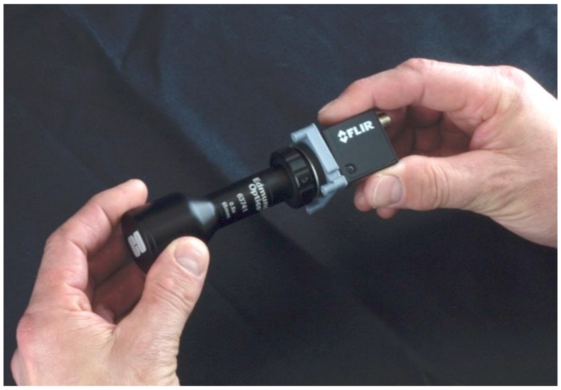

5. Seat the *camera* (8) in the **camera shoe (6)**. It is best if you use the mounting face of the *camera* (the face with the screw holes) for this – the mounting holes in the **camera shoe (6)** will align with the threaded holes in the *camera (8).*

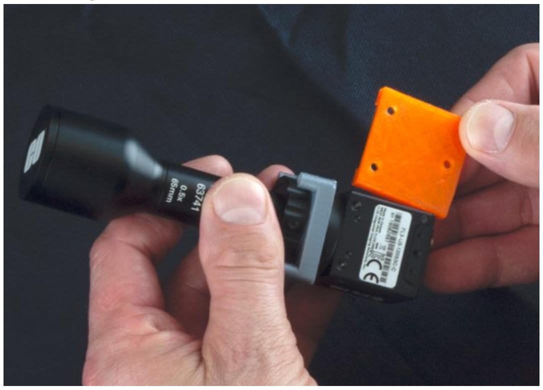

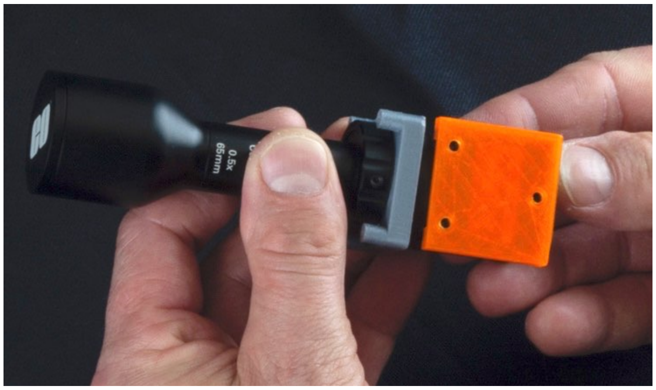

6. Remove the lens cap from the *lens (11).*

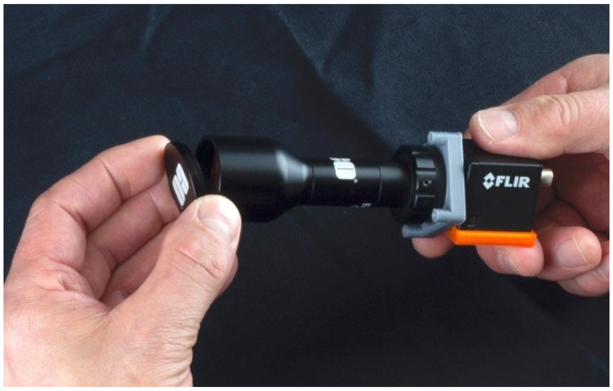

Conventions: 3D printed part names are in **bold case**. Other distinct parts are in *italics*.
Assemblies containing one or more parts of each type are shown in ***italics and bold***.

7. Place the ***lens-camera-Flea ring assembly*** on a flat surface, **camera shoe (6)** side down. Begin to slide the **lens ring (3)** onto the body of the *lens (11)*, taking care not to touch the lens' optical surface. When the **lens ring (3)** is installed it does not rotate (it is held in place by friction), so its orientation with regard to the *camera (8)* body and the **Flea ring (5)** is critical, as its flat sides must be square to the flat sides of the camera. This is necessary so that in later steps the ***camera-lens-rods-shoe assembly*** can slide into the *stainless steel tube (18).*

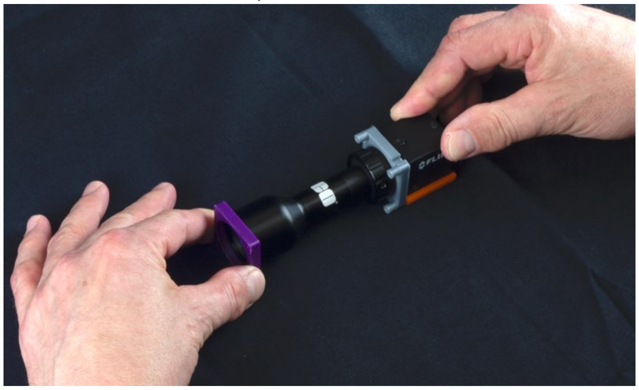

8. Use the **camera shoe (6)** and the flat surface to ensure that as the outer lip of the *lens (11)* tube is appressed to the stop ring on the inner surface of the **lens ring (3) -** any flat side of the **lens ring (3)** must be exactly parallel to the *camera (8)* body seated in the **camera shoe (6)**. The **Flea ring (5)** rotates freely, so its position at this point does not matter.

9. Lightly screw the lens cap back onto the *lens (11).*

10. Feed the four *rods (17)* through the holes in the **lens ring (3)** and into the posts in the **Flea ring (5)**. At the time of final assembly, it will be essential that the slot in the side of the **Flea ring (5)** is immediately adjacent to the **camera shoe (6)** – this is the slot through which the *barrel jack to 2 pin power cord (22)* will travel. This orientation will also ensure that the three through-holes for wiring the *switch (15)* will be on the non-mounting side of the *camera (8)*.

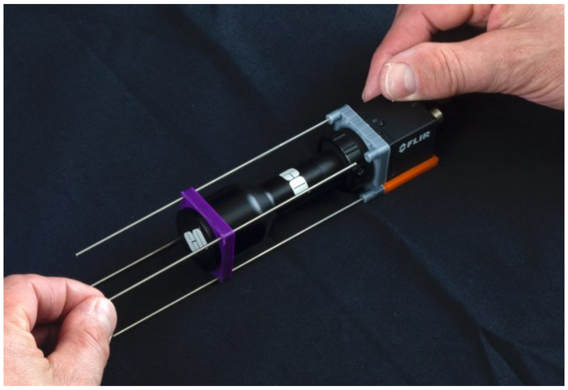

Conventions: 3D printed part names are in **bold case**. Other distinct parts are in *italics*.
Assemblies containing one or more parts of each type are shown in ***italics and bold***.

11. Lightly screw the **camera shoe (6)** to the camera body using one or more *camera mounting screws (19d, 19e)*. The lone hole at the back of the *camera (8)* is shallower than the two at the front – be sure to use the *6mm camera mounting screw* (19e) in this position and *8mm camera mounting screws (19d)* in the two forward holes.

12. Set aside the ***camera-lens-rods-shoe assembly*** for now

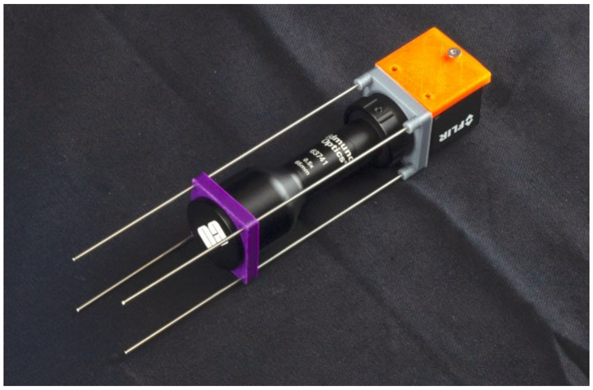

ELECTRONIC COMPONENTS – SOLDERING AND WIRING, PHASE I

Power input assembly

13. Clamp the *LED controller (12)* in a vise, pins-side up

14. Fit the *power output PCB (27)* onto the two pins on the *LED controller (12)*. The *power output PCB (27)* has a partial rectangle printed to show where the controller seats – that printed side will be in contact with the *LED controller (12)*, so there is only one way this *board* can be placed (note that the below left we are showing the face that will be in contact with the *LED controller (12)*. Below right we show the correct installation onto the two pins of the *LED controller (12)*. Solder the two pins, then flush cut them.

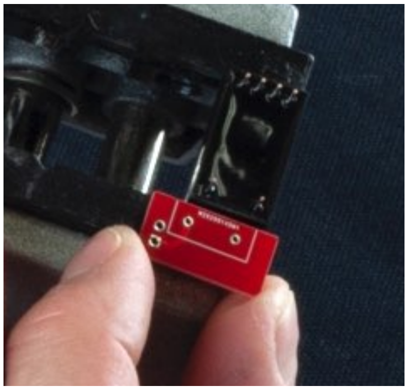
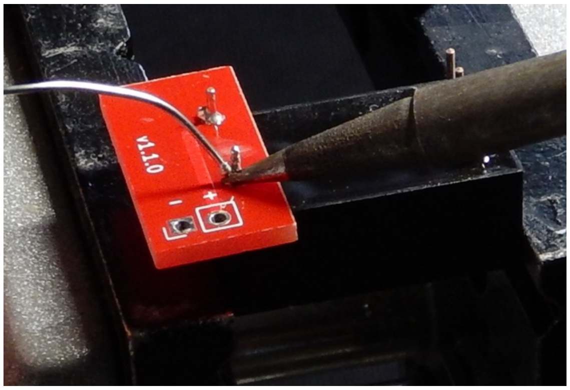

Conventions: 3D printed part names are in **bold case**. Other distinct parts are in *italics*.
Assemblies containing one or more parts of each type are shown in ***italics and bold***.

15. Fit the *power input PCB (26)* onto the four pins on the *LED controller (12)*. As with the *power output PCB (27)*, the *power input PCB (26)* has a partial rectangle printed to show where the *LED controller* seats (note in the image below-left: we are showing the face that will be in contact with the *LED controller (12)*. Below-right: we show the correct installation onto the four pins of the *LED controller (12)*. Solder all four pins, then flush cut them.

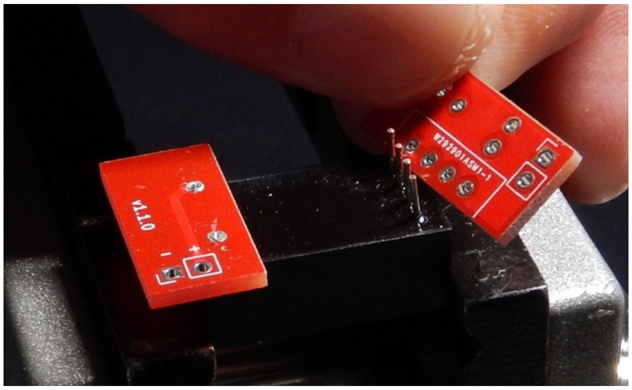

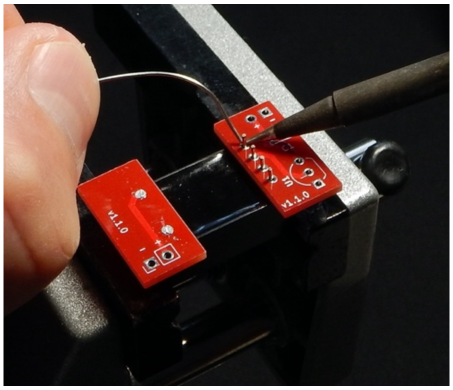

16. Insert the *voltage reference (20)* into the three holes in the *power input PCB (26)*. There is a semicircular print with a flat edge associated with the three mounting holes on the board. Install the *voltage reference (20)* so that its flat face is facing the flat side of the shape outline. Gently and carefully bend the leads so that the bottom of the *voltage reference (20)* can be mounted within 2-3mm of surface of the *power input PCB (26)*. Solder the three leads and flush cut them.

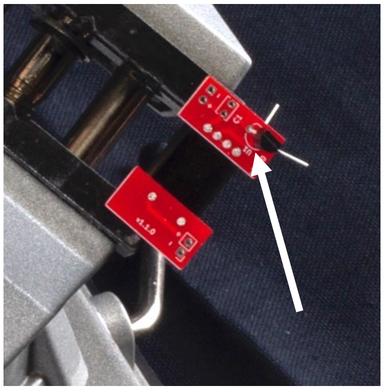

17. Insert the *radial capacitor (13)* into the two holes in the small printed rectangle so that its height is less than that of the *voltage reference (20)*. There is no special orientation for the *radial capacitor (13)*. Solder and flush cut.

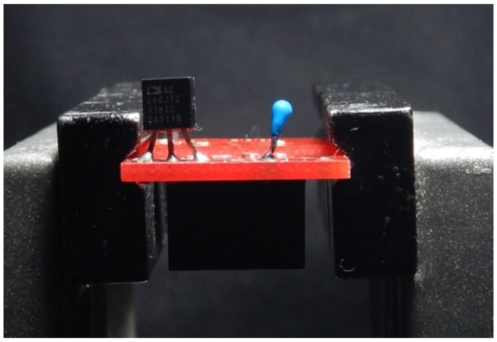

18. The last component of the *power input PCB (26)* is the *right angle JST through-hole connector (16)*. This fitting is inserted from the *LED controller (12)* side of the *power input PCB (26)* so that its pins emerge by the printed – and + near the *radial capacitor (13)*. Solder these two pins, taking care to make mechanically strong connections with the board without angling the pins or melting through to the plastic housing. Flush cut the pins.

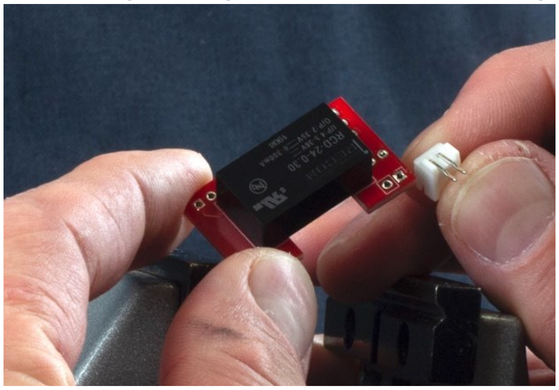
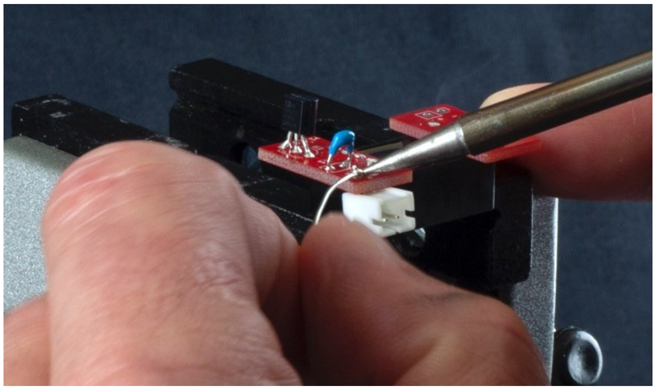

19. Insert the JST male end of the *barrel jack to 2 pin power cord (22)* into the *right angle JST through-hole connector (16)* that was just soldered. The *power input assembly* of the XyloTron is now complete. Set aside this assembly.

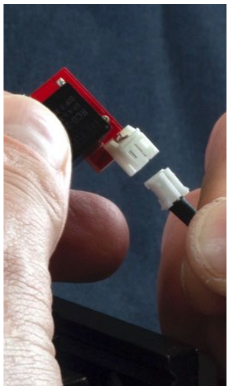
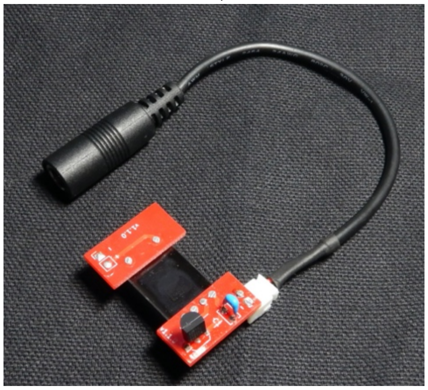
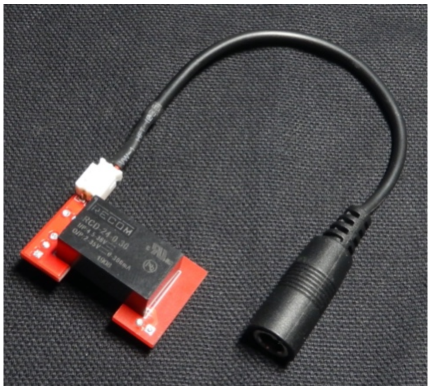

Conventions: 3D printed part names are in **bold case**. Other distinct parts are in *italics*.
Assemblies containing one or more parts of each type are shown in ***italics and bold***.

VIS LED-LED PCB assemblies

20. Clamp an *LED PCB (28)* in the vise. It is easiest to grip the narrow end of the *LED PCB (28)* that contains the two through holes, so that the soldering pads face you. Position the *VIS LED (14)* on the soldering pads with the small triangular notch on the lower right (facing the dot) – this places it on the negative pad, nearest the wide edge of the *board*. Do not let the *VIS LED (14)* protrude past that wide edge of the *board* – it is best if it is set back from the edge about 0.5mm*. Solder the *VIS LED (14)* in place on both pads.

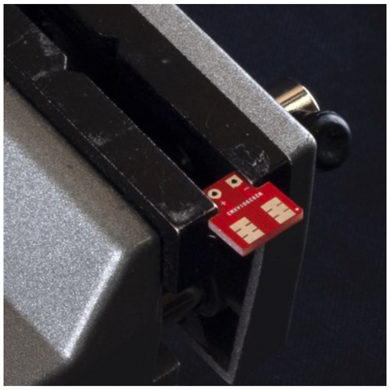
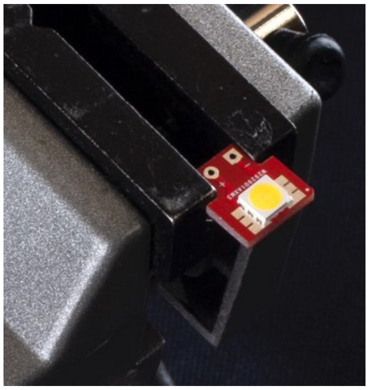
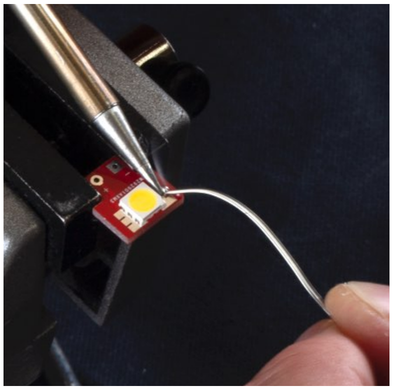

**Doing this can be a challenge. A useful hack involves pinching the VIS LED (14) onto the board with flat tweezers, and then using a small binder clip to holder the tweezers in place. The compliance of the tweezers ensures that excessive force is not applied but permits fine spatial adjustment of the VIS LED (14) position. This assembly is then clamped in the vise. Heating the soldering pads without touching the VIS LED (14) and then allowing capillary flow to make the connection is usually successful.* Repeat this process three more times.

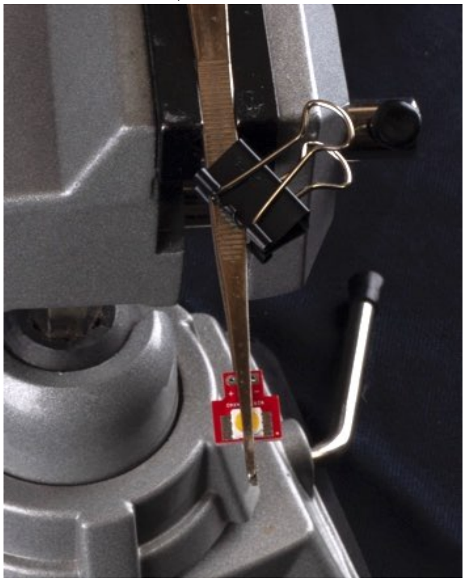
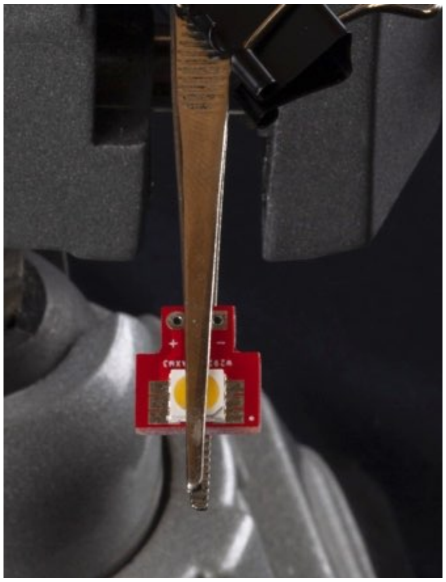

21. Using an LED testing setting on a multimeter, ensure that each *VIS LED-LED PCB assembly* lights <u>both</u> internal LEDs in the *VIS LED (14)*. This is best done at the +/- through holes, to ensure the entire assembly is electrically correct.

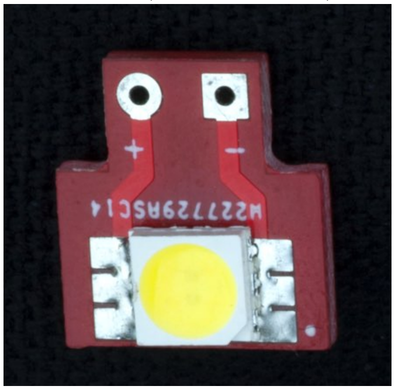

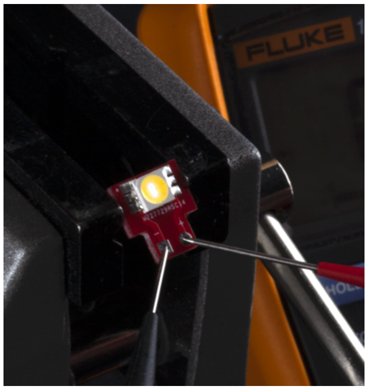

Power distribution board-UV LED assembly

22. Clamp the *power distribution PCB (25)* in the vise, with side with the printed "UV" "VIS" "NC" and "GND" facing up. Place one *press-fit receptacle (21)* in each of the four-corner plated through-holes. Solder each in place, taking care to keep the bottom of the hexagonal head of the fitting flush with the board and ensuring that no solder flows over the lip and into the fitting.

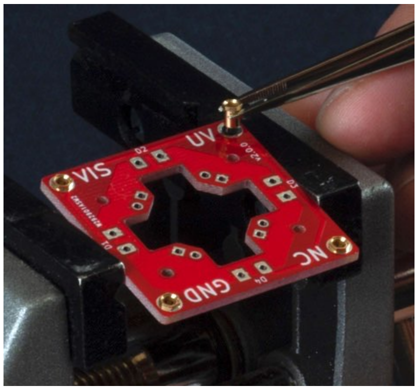

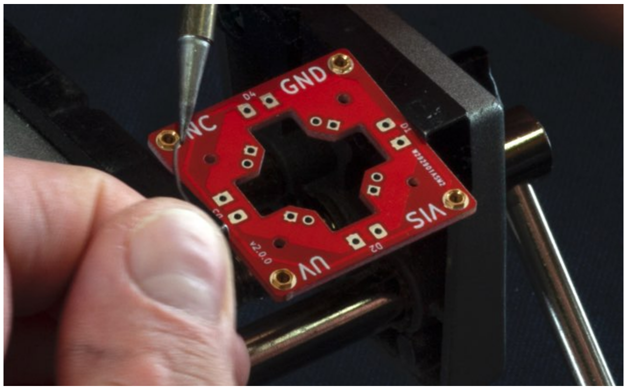

23. Flip over the *power distribution PCB (25)* and observe the pairs of through holes labeled D5, D6, D7, and D8. This is where you will solder the *UV LEDs (10)*. Clamp the *power distribution PCB (25)* in the vise, with side with the printed "UV" "VIS" "NC" and "GND" facing up. If you are right-handed then fill the left (and only the left) of the two through holes at D5, D6, D7, and D8 with solder. There should be a slightly (but only slightly) raised meniscus of cooled solder in each through hole on the opposite face of the *power distribution PCB (25)*.

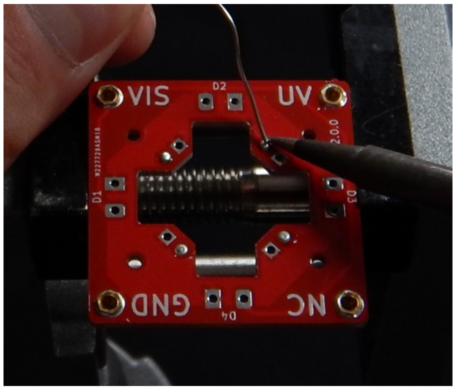

THE NEXT TWO STEPS ARE THE MOST DIFFICULT OPERATIONS IN THE ASSEMBLY OF THE XYLOTRON

24. Flip over the *power distribution PCB (25)* so that the printed "UV" "VIS" "NC" and "GND" side is facing up. Using fine tipped tweezers, orient the *UV LED (10)* so that its positive pad corresponds to the + through hole at D5, D6, D7, or D8 and so that you can press it flush to the face of the through hole. Get a tiny bead of melted solder on the tip of the soldering iron and use that that to melt the solder in the through hole. Lower the *UV LED (10)* in place, gently press it onto the through holes of the *power distribution PCB (25),* and then remove the soldering iron. When the solder cools, one of the two pads of the *UV LED (10)* will be soldered.

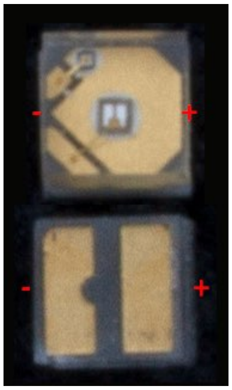

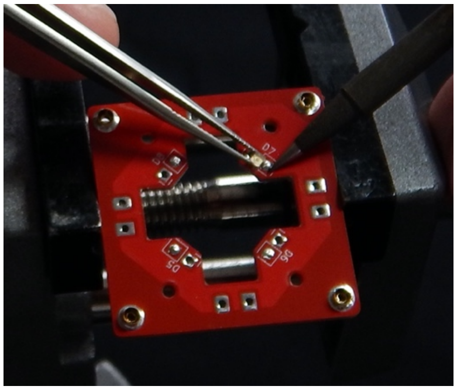

25. Place the solder at an angle into the through hole adjacent to the other pad on the *UV LED (10)*, and WITHOUT TOUCHING THE LED WITH THE SOLDERING IRON, solder the other pad of the *UV LED(10)*. Repeat these steps for the other three *UV LEDs (10)*. The *power distribution board-UV LED assembly* is complete for now.

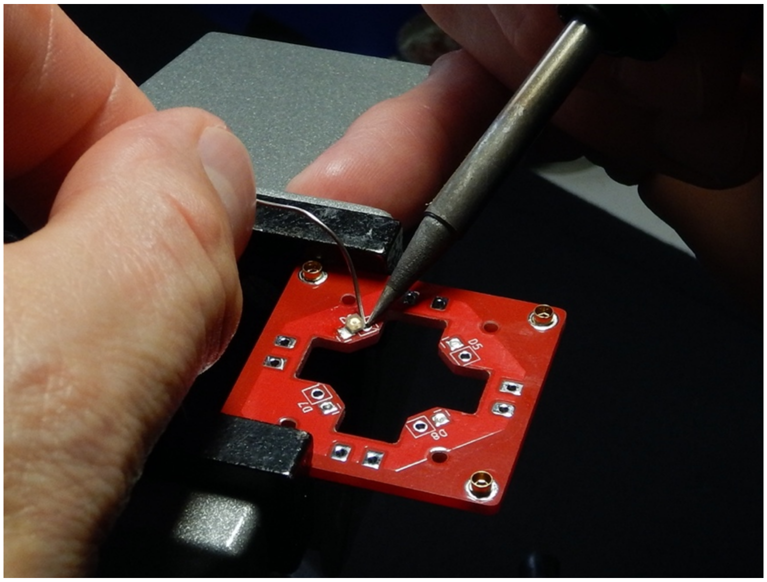

26. Before proceeding, it is a good idea to confirm that your soldering work is sound and electrical connections are made correctly. This is most easily done by using the *power input assembly* completed in Step 19 to power the *power distribution board-UV LED assembly*. Plug the *barrel jack to USB adapter cord (24)* into a 5V DC power source (a cell phone charger is a convenient source of 5V DC power), and then connect the *two-barrel connectors (22* and *24)*. There is only one way this will work – it will be obvious.

27. Using the *JST jumper two wire assembly (23)*, push the pins through the through holes on the *power output PCB (27)* ensuring that the red wire is connected to the + through hole and the black wire is connected to the - through hole.

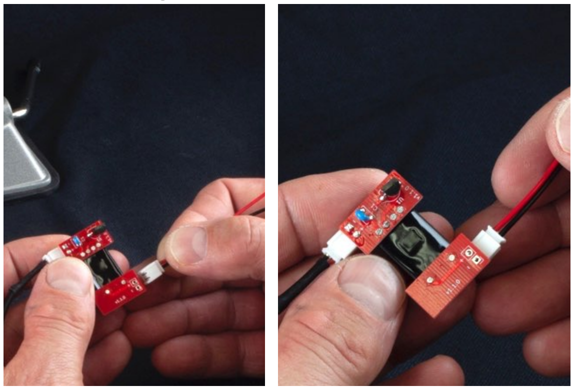

Conventions: 3D printed part names are in **bold case**. Other distinct parts are in *italics*.
Assemblies containing one or more parts of each type are shown in ***italics and bold***.

28. Ensuring that the pins of the *JST jumper two wire assembly (23)* are in contact with the through holes of the *power output PCB (27)*, touch the black (-) wire to the GND corner's *press-fit receptacle (21)* – the one near D5 – and the red (+) wire to the UV corner's *press-fit receptacle (21)* – the one near D7 – on the *power distribution board-UV LED assembly*. If all electrical connections are correct, all four *UV LEDs (10)* will light up. Remove the *JST jumper two wire assembly (23)* if all is well. If at least one *UV LED (10)* lights, then you know that your *press-fit receptacle (21)* connections for GND and UV are sound, and you can work to re-solder the unlit *UV LEDs (10)*.

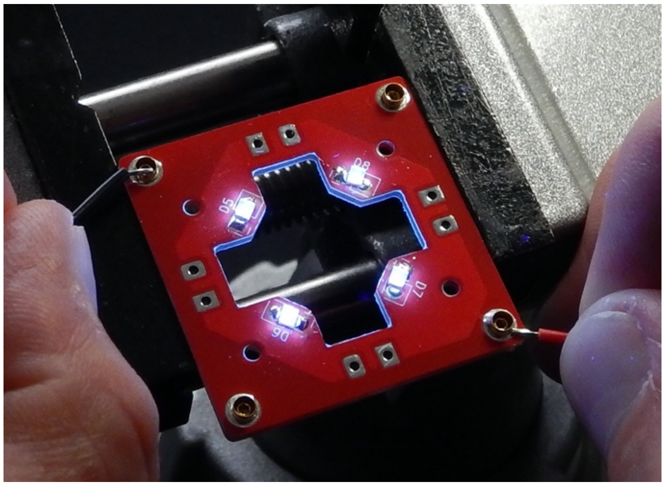

Mechanical assembly – phase II

LED holder-power distribution board assembly

29. Now that the *UV LEDs (14)* are soldered onto the *power distribution board-UV LED assembly* it is important that as you handle the *assembly* you do not damage the *UV LEDs (14)*. They are well-suited to thousands of hours of data collection, but are not resilient in the face of mechanical damage. As you place the *assembly* do not touch or break off the *UV LEDs (14)*. Fit the *power distribution board-UV LED assembly* onto the flat surface of the **LED holder (2)**. There are small tabs into which the protruding cylindrical portions of the *press-fit receptacles (21)* will fit – sometimes it is necessary to clean out the printed holes so that the *power distribution board-UV LED assembly* will sit flush. This can be accomplished with a sharp, small-bladed knife. Use four *¼" #0 thread-forming screws (19c)* to mount the *power distribution board-UV LED assembly* to the **LED holder (2)** – there are only two orientations where the screw holes will mate to the holes in the **LED holder (2)**, in order to make the ***LED holder-power distribution assembly.***

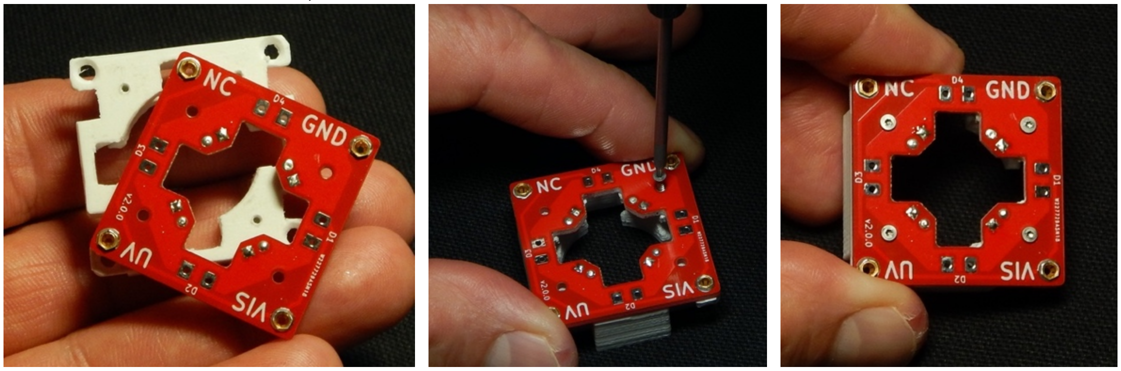

Conventions: 3D printed part names are in **bold case**. Other distinct parts are in *italics*.
Assemblies containing one or more parts of each type are shown in ***italics and bold***.

30. Insert the four *VIS LED-LED PCB assemblies* into the ***LED holder-power distribution assembly***. When they are fully seated, the through holes at the narrow end of the *VIS LED-LED PCB assemblies* will be roughly flush with the *power distribution assembly* on the ***LED holder-power distribution assembly***.

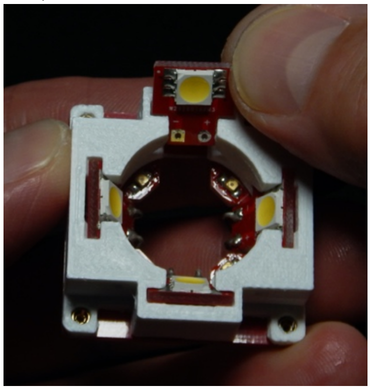

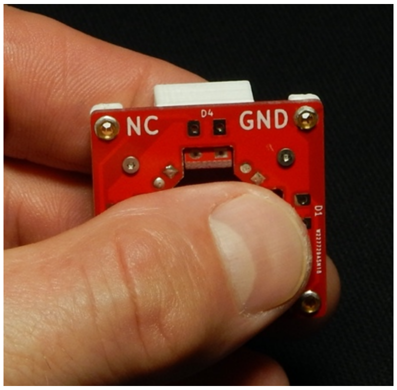

ELECTRONIC COMPONENTS – SOLDERING AND WIRING, PHASE II

LED holder-power distribution board assembly

31. Using short lengths of wire (e.g. 26 ga. braided insulated copper) connect the + through hole at D1, D2, D3, D4 to the + through hole on the *VIS LED-LED PCB assemblies*. Strip part or all the insulation off the wire, twist, and tin the wire if needed to prevent the end from fraying when you feed it through the + through hole on the *power distribution PCB* of the ***LED holder-power distribution assembly***. Tweezers help threading the wires. Repeat for the – through holes. Solder the wires in place and flush cut any excess. You can use short lengths of wire and enter the *VIS LED-LED PCB* from the back side of the PCB (as shown immediately below) or you can use longer wires and enter the through holes from the front of the *VIS LED-LED PCB* PCB. It is tidied to enter from the back, but either way works. The ***LED holder-power distribution board assembly*** shown in the rest of the manual has three *VIS LED-LED PCBs* wired from the front and one from the back.

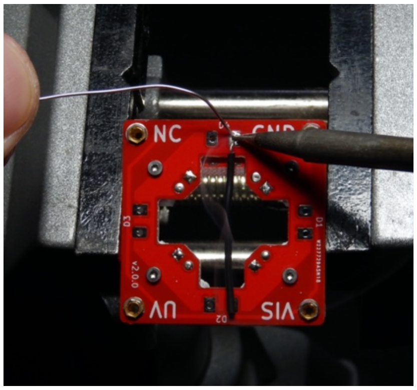

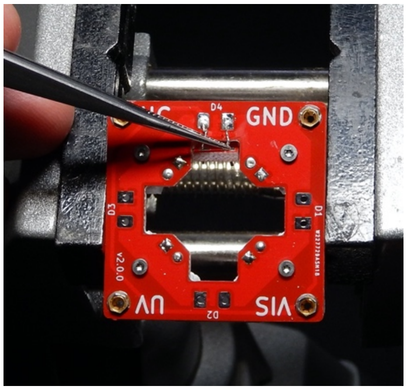

32. As with Steps 26-27, it is prudent (and satisfying) to confirm that the electrical connections are sound. Using the same assembly in Steps 26-27, and ensuring that the pins of the *JST jumper two wire assembly (23)* are in contact with the through holes of the *power output PCB (27)*, touch the black (-) wire to the GND corner's *press-fit receptacle (21)* and the red (+) wire to the VIS corner's *press-fit receptacle (21)* on the *power distribution board-UV LED assembly*. If all electrical connections are correct, all four *VIS LEDs(14)* will light up. Remove the *JST jumper two wire assembly (23)* if all is well. If at least one *VIS LED* lights, then you know that your *press-fit receptacle (21)* connections for GND and VIS are sound, and you can work to re-solder the unlit *VIS LED-LED PCB assemblies'* connections to the *power distribution board-UV LED assembly* (because you already confirmed each *VIS LED-LED PCB assembly's* electrical correctness in Step 21).

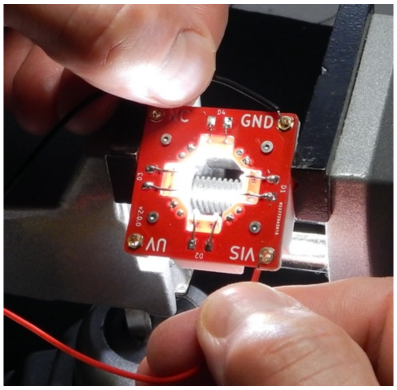

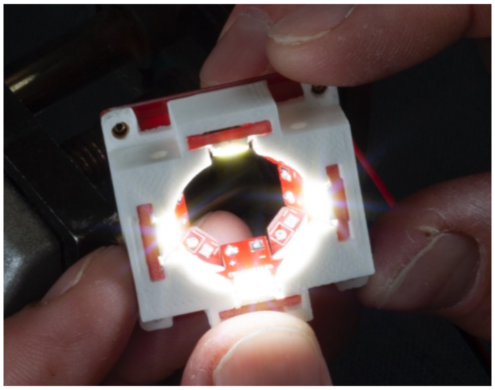

33. Fit three *press-fit receptacles (21)* onto a short length of spare *rod (17)* (child = spoiled) and prepare two insulated copper wires, approximately 15cm in length each, and one approximately 5cm in length, each of a different color. If you have no spare *rod (17),* remove one of the *rods (17)* from the ***camera-lens-rods-shoe assembly*** and use that, then replace it when done. Strip several mm of insulation from the end of each wire and twist the ends. Slide one *press-fit receptacle (21)* to the end of the *rod (17)* and secure it in the vise. Using a spot of solder, fix the very tip of the wire to the outer cylindrical surface of the *press-fit receptacle (21)*, allow the solder to cool, then tightly wrap the remaining bare wire around the circumference of the cylindrical portion of the *press fit receptacle (21)* and solder this. It is important that the wire plus solder not be too bulky, as these *press-fit receptacle and wire assemblies* must slide into the printed holes in the **sliding LED controller carriages (4)**. It can require a few attempts to develop a technique that results in a tight wrap. Repeat this for the remaining two wires.

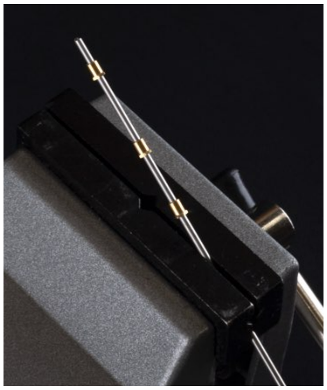
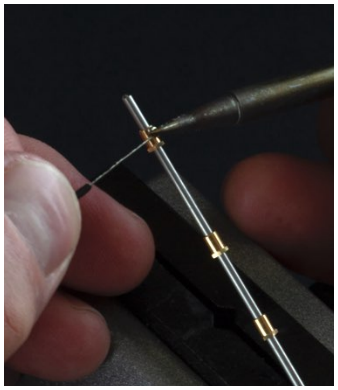
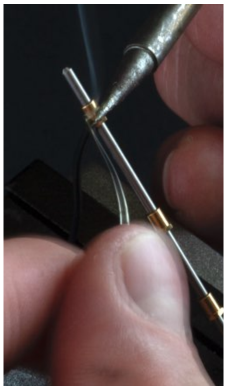
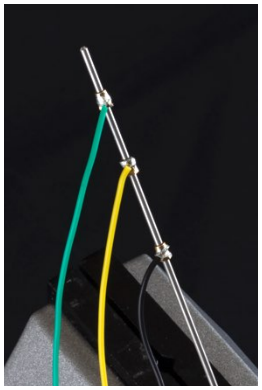

INTEGRATING ELECTRONIC AND MECHANICAL COMPONENTS

Full XyloTron internal assembly

34. Slide the ***LED holder-power distribution-LEDs assembly*** onto the *rods (17)* with the *LEDs* facing away from the *lens (11)* and oriented so that the “UV” and “VIS” corners are oriented opposite the mounting side of the *camera (8)* – this is the only orientation that will function, as the *rods (17)* will conduct the electricity to power the ***LED holder-power distribution-LEDs assembly***. It can require a careful, gentle tap to force each *rod (17)* into a *press-fit receptacle (21)* the first time.

35. Slide the **front cap (1)** onto the *rods (17)*. This helps stabilize the ***assembly*** for now. Ensure that the **front cap (1)** is rotated so that the **LED holder (2)** can mesh with the **front cap (1)**. This will also ensure that mounting holes in the **front cap (1)** and **LED holder (2)** are aligned with the corresponding holes in the *stainless steel tube (18)*.

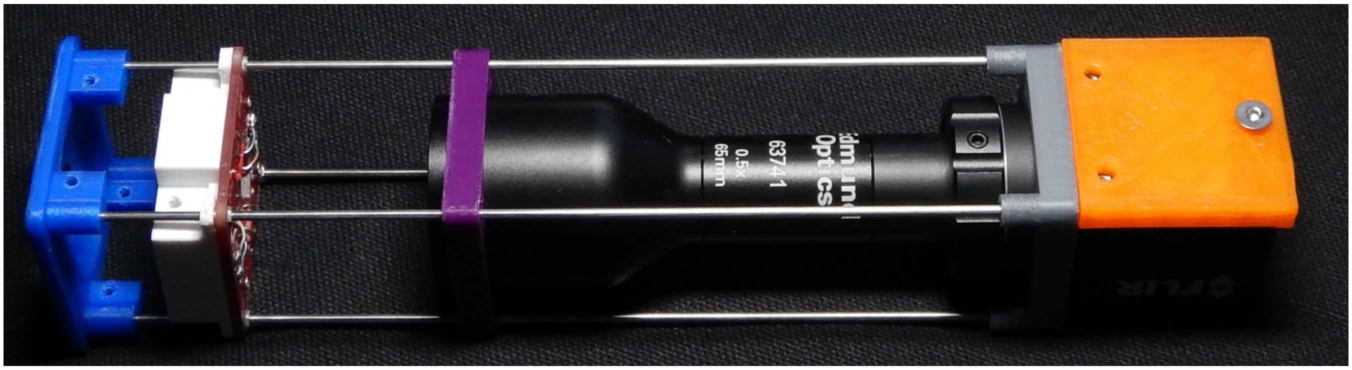

36. From the **LED-holder (2)** side of the assembly (left side in the image below), back the *rods (17)* out of the **Flea ring (5)** so that there are 2-3 cm of space between the ends of the *rods (17)* and the **Flea ring (5)** posts.

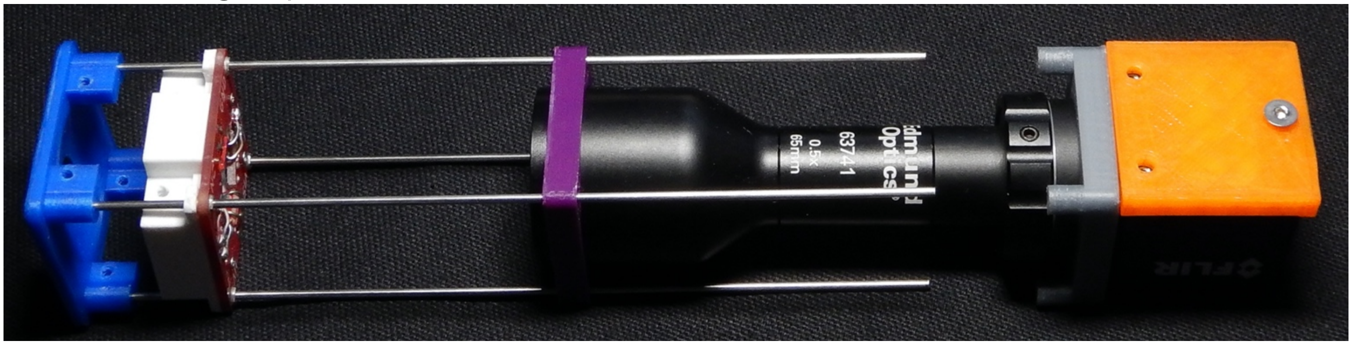

37. Fit one **sliding LED controller carriage (4)** over the body of the *LED controller (12)* portion of the *power input assembly* – ensure that the cut-out for the *right angle JST through-hole connector (16)* fits into the printed slot. This is easier if you remove the *barrel jack to 2 pin JST power cord (22)*. Slide this ***assembly*** onto the two *rods (17)* forming a plane parallel to the mounting side of the *camera (8)* – this will be spanning "GND" and "NC" on the *power distribution board*. In doing this, the *barrel jack to 2 pin JST power cord (22)* will fit into the slot on the **Flea ring (5)**, and travel alongside the *camera (8)* body just under the lip of the **camera shoe (6)**. The printed *power input PCB* and the *power output PCB* will face into the body of the XyloTron, toward the *lens (11)* tube.

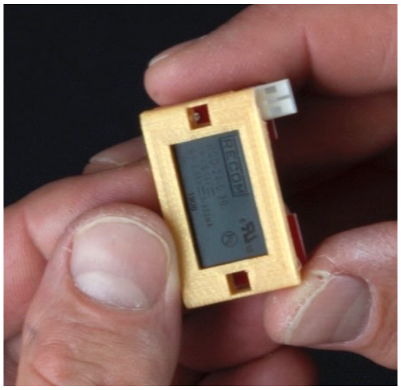

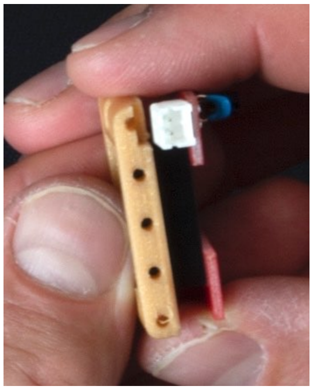

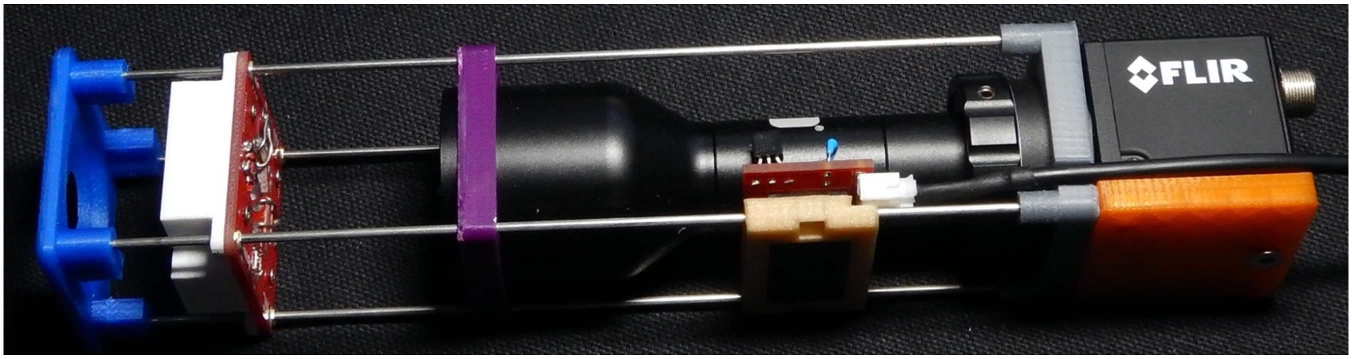

38. After ensuring that the relationships in the previous step are correct, back the **sliding LED controller carriage (4)** off the *rods (17)* until it is possible to fit the short wired *press-fit receptacle and wire assembly* into the printed hole in the **sliding LED controller carriage (4)** corresponding to the "GND" *rod (17)*, with the hexagonal end of the *press-fit receptacle (21)*, facing away from the *camera (8)* and toward the *lens (11)* – the wire should exit the slot toward the *lens (11)* tube (toward the center of the device). Slide the *rod (17)* through the *press-fit receptacle and wire assembly*, and out through the **sliding LED controller carriage (4)** and into the corresponding **Flea ring (5)** post. Do the same with the unused *rod (17)* passing through the **sliding LED controller carriage (4)**.

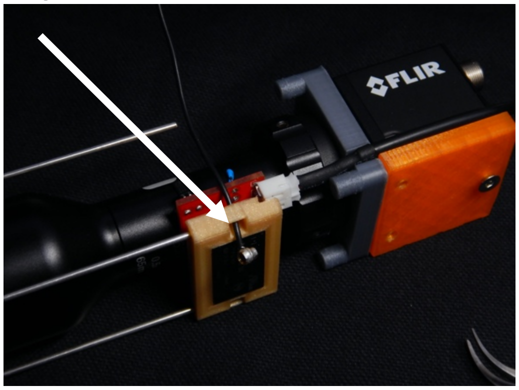

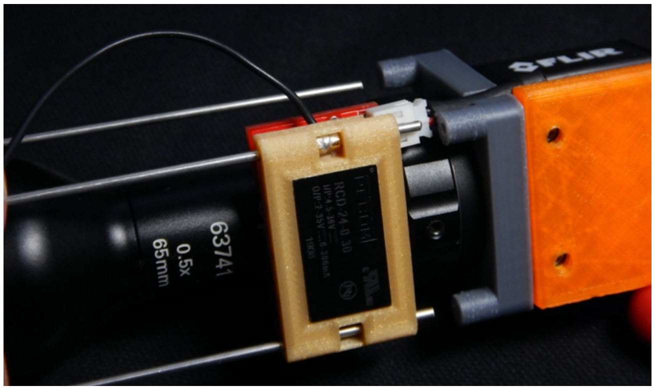

39. The second **sliding LED controller carriage (4)** will be installed spanning the "UV" and "VIS" *rods (17)*. Install the two longer wired *press-fit receptacle and wire assemblies*, one on each *rod (17)*, as in the previous step. Thread the loose end of each wire through the nearest **sliding LED controller carriage (4)** through-hole toward the *camera (8)*, and then through the corresponding through-hole in the **Flea ring (5)**. These two wires should protrude past the back end of the *camera (8)* by several centimeters. In the configuration below, the yellow wire will power the VIS *rod (17)* and the green wire will power the UV *rod (17)*.

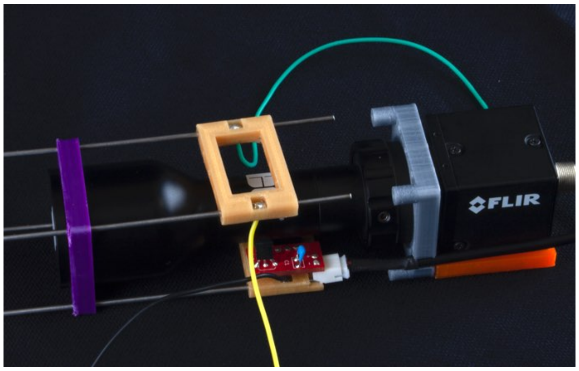

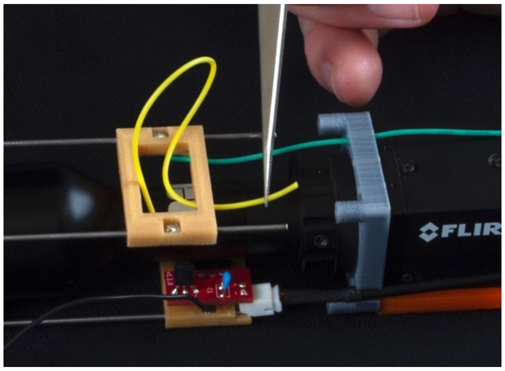

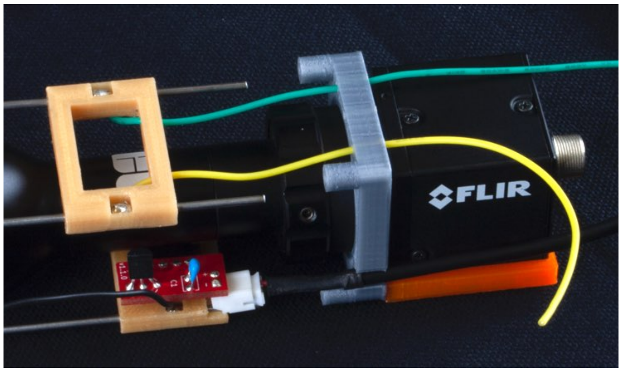

Conventions: 3D printed part names are in **bold case**. Other distinct parts are in *italics*.
Assemblies containing one or more parts of each type are shown in ***italics and bold***.

40. Cut a 15 cm length of wire and strip and twist one end. Insert it into the + through hole on the *power output PCB (27)*, solder it in place, and flush-cut it. Thread the other end of the wire through the central through-hole in the **sliding LED controller carriage (4)** through which the other two *wires* were routed, then through the center hole in the **Flea ring (5)**. This wire, red in the case of the images below, carries power from the *power output PCB (27)* to the center terminal of the *switch (15)*. When the *switch (15)* is in the central position, no current flows.

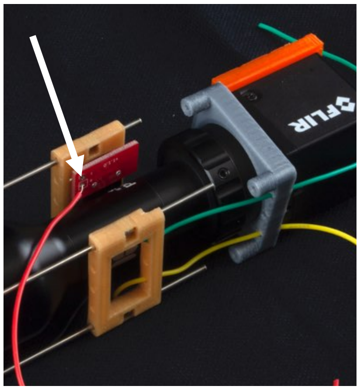
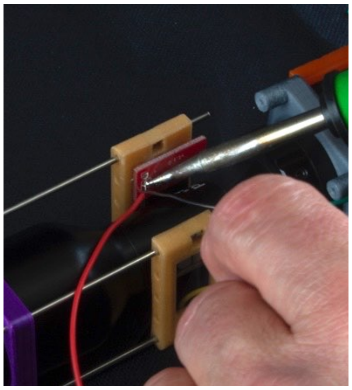
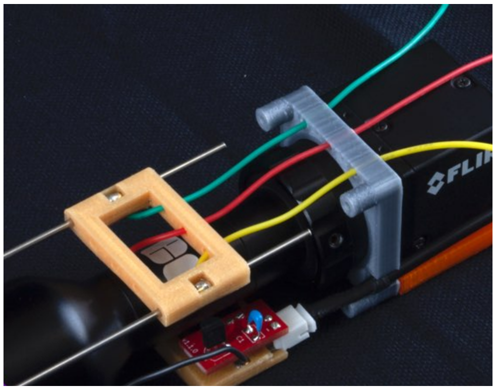

41. It can be helpful to use a piece of transparent tape folded over on itself to sandwich the three loose wires as they exit the **Flea ring (5)** so that as a group they lay flat and do not bunch up under the *camera (8)*. Re-seat the *rods (17)* firmly in the posts of the **Flea ring (5)**.

42. Fish the end of the short wire from the "GND" rod *press fit receptacle wire assembly* to the *power output PCB (27)*, strip the end of the wire, solder to the – through hole, and flush cut.

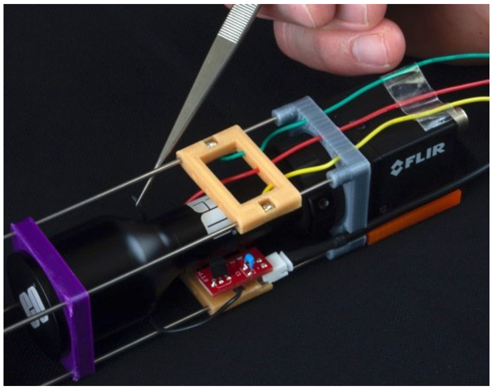

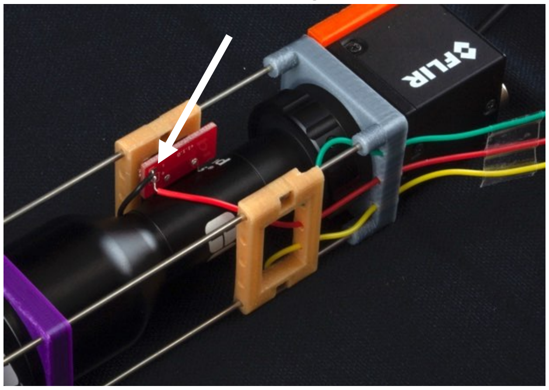

43. Insert the *switch (15)* into the **back cap (7)** and screw into place with two *¼" #0 thread-forming screws (19c)*.

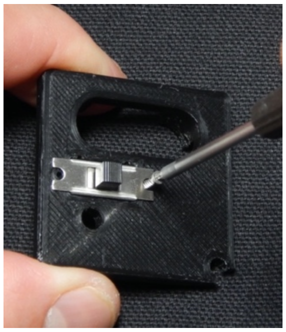

Conventions: 3D printed part names are in **bold case**. Other distinct parts are in *italics*.
Assemblies containing one or more parts of each type are shown in ***italics and bold***.

44. Determine the length of wire necessary to connect to the terminals on the *switch (15)* and still allow the **back cap (7)** to be maneuvered as the internals are placed in the *stainless steel tube (18)*. Leaving the wires too long will result in more difficult wire management.

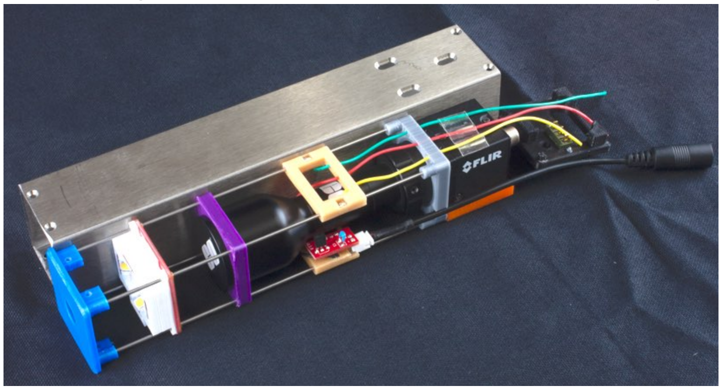

45. Remove the *camera mounting screw(s) (19d, 19e)* holding the **camera shoe (6)** in place. Remove the lens cap. Position the ***LED holder-power distribution board assembly*** in whichever illumination position along the *rods (17)* you desire. The default (wood) position would be mated fully with the **front cap (1)**.

Below are pictured the two basic configurations, the default (wood) position above, and the charcoal position below.

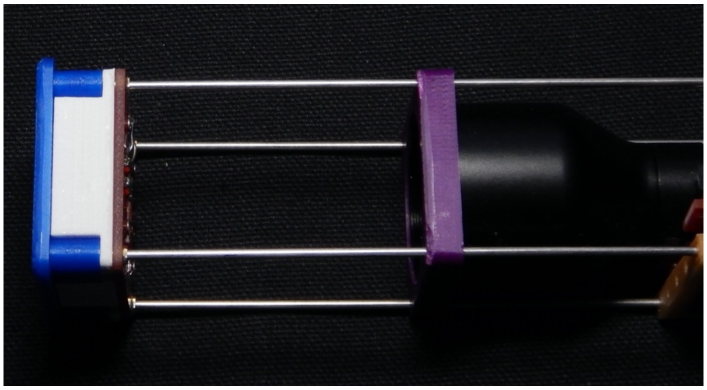

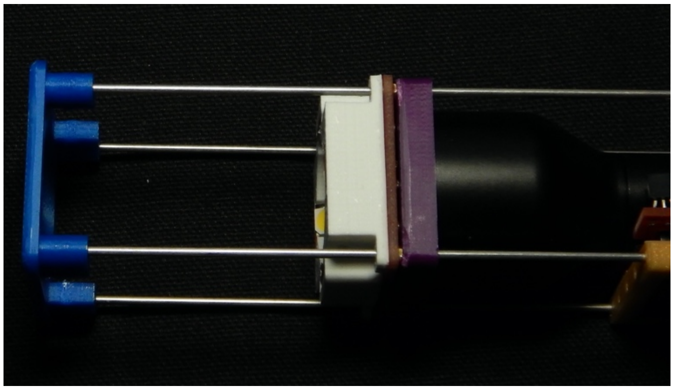

Conventions: 3D printed part names are in **bold case**. Other distinct parts are in *italics*.
Assemblies containing one or more parts of each type are shown in ***italics and bold***.

46. Slide the ***full XyloTron internal assembly*** into the *stainless steel tube (18)* from the front end taking care to ensure that wires are not pinched between internal components and the lip of the *tube*. When the *camera's* mounting holes align with the slots in the *tube*, secure the ***full XyloTron internal assembly*** in place with one or more *camera mounting screws (19d, 19e)*.

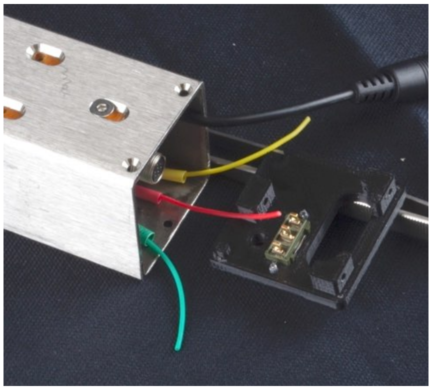

47. Slide one piece of shrink tubing up each of the three wires – do not let them come too close to the *switch (15)* or the heat of soldering will cause premature shrinkage. Strip the end of the wire connected to the + terminal of the *power output PCB (27*)(red in the images here), twist, pull it through the center terminal on the *switch (15)*, mechanically twist it to the terminal, then solder in place. Repeat this wire stripping and soldering for the remaining two wires (one yellow "VIS" and one green "UV") to the remaining two lateral terminals. Allow the wires and posts to cool. Slide the shrink tubing down over the cooled, soldered terminals. Carefully shrink the tubing with a heat gun – the heat gun can easily melt the **back cap (7)**, so care is needed.

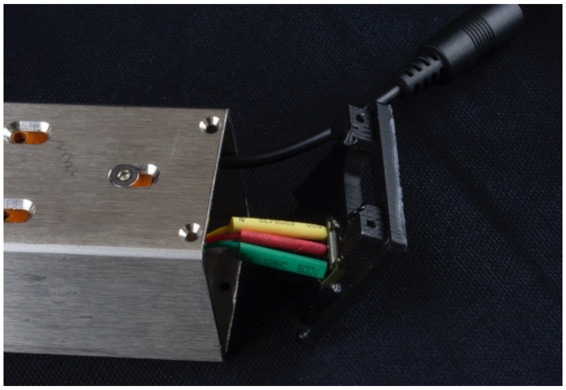

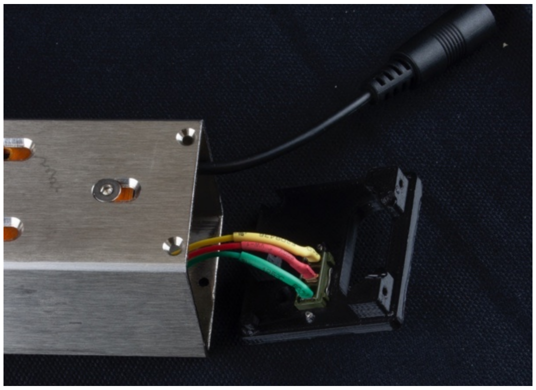

48. Using three *¼" #2 thread-forming screws (19b)*, secure the **back cap (7)** to the *stainless steel tube (18)*. Take care to ensure that the *switch (15)* wires are tucked along the *camera (8)* body and do not block the connection for the *USB 3 cable (9)* to the *camera (8)*.

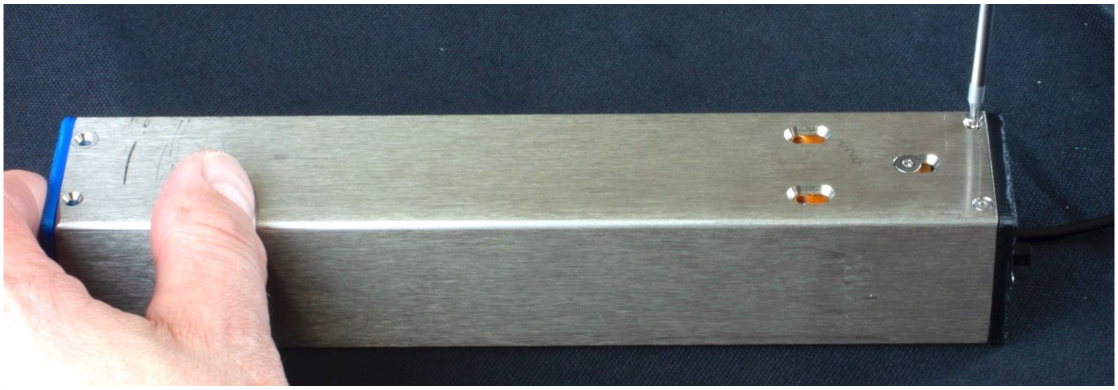

49. Secure the **front cap (1)** to the *stainless steel tube (18)*. If you want to maintain the ***LED holder-power distribution board assembly*** in the modular configuration, use four short *¼" #2 thread-forming screws (19b)*. If you wish to fix the ***LED holder-power distribution board assembly*** in the wood configuration, mate the **front cap (1)** snugly to the ***LED holder-power distribution board assembly*** and then use at least one longer *½" #2 thread-forming screw (19a)* per face. If not already done, screw in the remaining *camera screws (19d, 19e)*. This completes the mechanical and electronic systems of the ***XyloTron***.

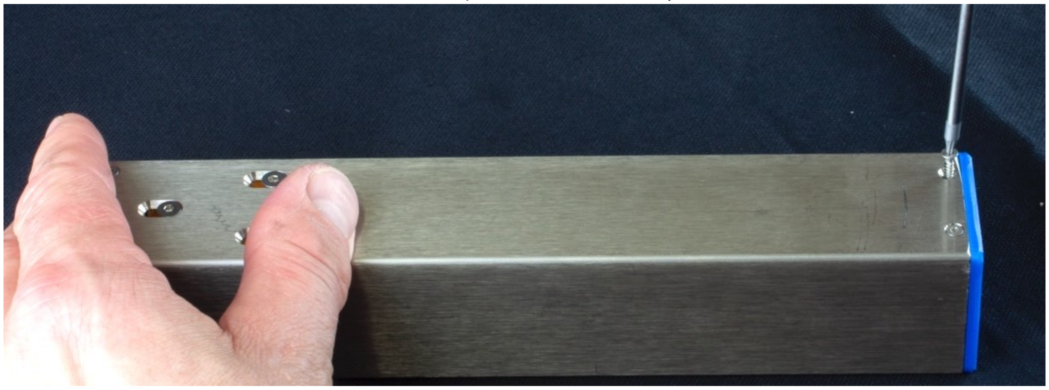

FOCUSING THE XYLOTRON

50. Connect the *USB3 cable (9)* and *USB barrel connector cable (23)* of the ***XyloTron*** to the computer and a 5V DC power source, respectively. (The latter can be a USB port on a computer). Ensure that the *USB3 cable (9)* is connected to a USB3 (not USB2) port on the computer. This process is independent of the lighting configuration or lighting source.

51. Open the FlyCap software (the FLIR website provides the installer). Click the "Camera Control Dialog" button to open the control window. Manually configure the camera settings as follows:
    a. Under the "Custom Video Modes" tab:
        i. Enter 2048 for both "Width" and "Height"
        ii. Click the "Center ROI" button
        iii. Click apply.
    b. Under the "Camera Settings" tab:
        i. Brightness = 0

ii. Exposure: unclick the Auto box, value 0.8
iii. Sharpness = 1024
iv. Hue = 0
v. Gamma = 1.00
vi. Shutter = Auto should be clicked
vii. Gain: unclick Auto box, value 0.00
viii. Frame rate: unclick Auto box, value 6.0
ix. Red and Blue values: for now do not adjust

52. Adjust the zoom value for the main FlyCap window so that the full field of view is visible. Maximize the size of the window to allow for the best possible viewing.

53. Lay the ***XyloTron*** down horizontally, and place an object of interest flush with the **front cap (1)**. Loosen the *camera mounting screws (19d, 19e)* so that the ***full XyloTron internal assembly*** can be slid back and forth in the *stainless steel tube (18)*.

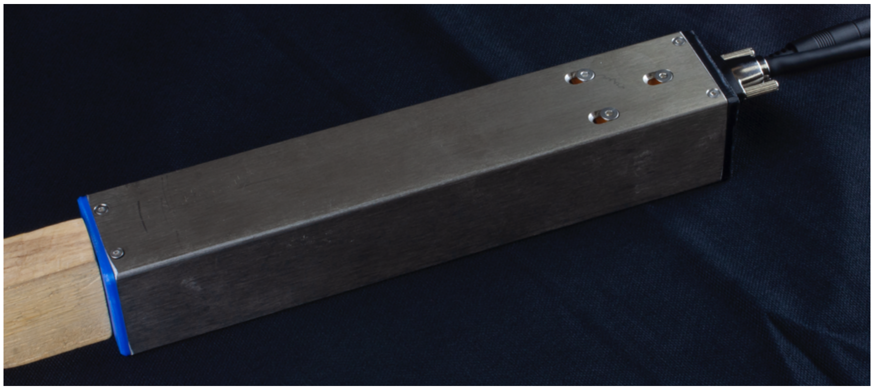

54. Using the *USB3 cable's (9)* locking mount, gently slide the ***internal XyloTron assembly*** forward and backward, keeping the object of interest firmly appressed to the **front cap**, observing how the object of interest goes in and out of focus. The ***XyloTron*** has about 500-700um of focal depth, and we want that focal plane to be mostly extending past the front of the **front cap (1)**, so that if the ***XyloTron*** is placed on an irregular surface (e.g. a piece of wood cut in the field with a utility knife) the surface of interest can be in focus. This can require some trial and error – take your time to do this precisely.

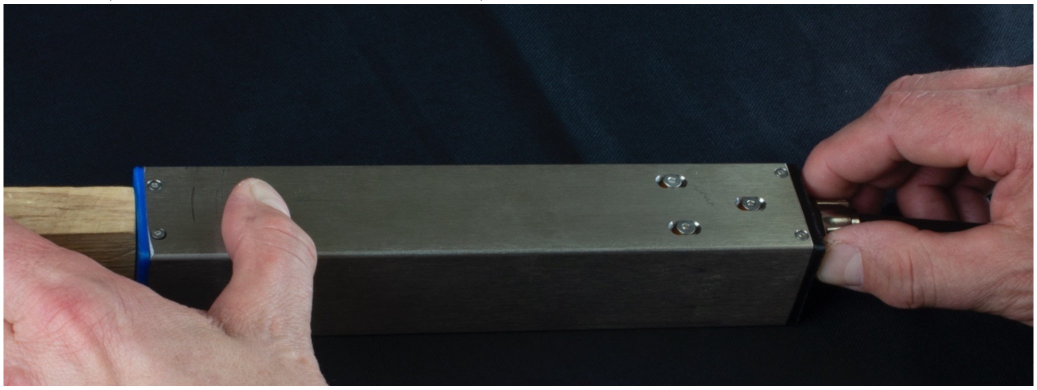

55. Once the focal distance is correct, tighten the rear-most *6mm camera mounting screw (19e)*. Slide the object of interest away from the front of the ***XyloTron***, ensuring that even when it is a small distance from the **front cap (1)**, it remains in focus. Revisit the prior step if tightening the *screw* shifted the focal position.

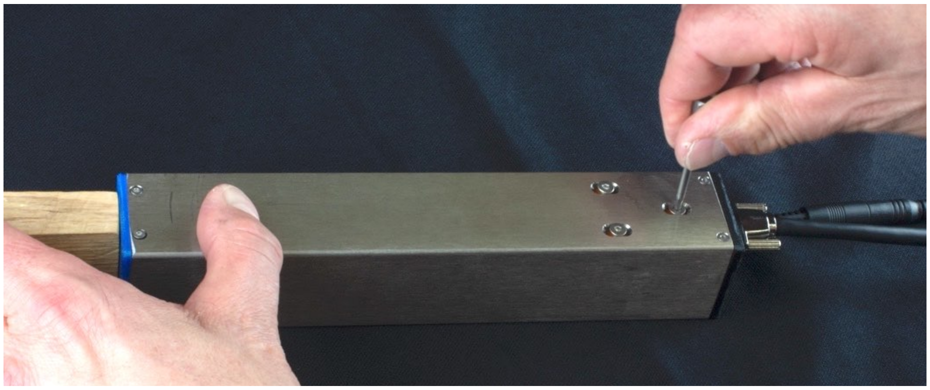

56. Tighten the remaining two *8mm camera mounting screws (19a)* firmly. The ***XyloTron*** is now focused.

WHITE BALANCING THE XYLOTRON

57. In not already done, connect the *USB3 cable (9)* and *USB barrel connector cable (23)* of the ***XyloTron*** to the computer and a 5V DC power source, respectively. (The latter can be a USB port on a computer). Ensure that the *USB3 cable (9)* is connected to a USB3 (not USB2) port on the computer. This process is independent of the lighting configuration or lighting source

58. Open the FlyCap software. Click the "Camera Control Dialog" button to open the control window. Manually configure the camera settings as follows:

a. Under the "Custom Video Modes" tab:
   i. Enter 2048 for both "Width" and "Height"
   ii. Click the "Center ROI" button
   iii. Click apply.
b. Under the "Camera Settings" tab:
   i. Brightness = 0
   ii. Exposure: unclick the Auto box, value 0.8
   iii. Sharpness = 1024
   iv. Hue = 0
   v. Gamma = 1.00
   vi. Shutter = Auto should be clicked
   vii. Gain: unclick Auto box, value 0.00
   viii. Frame rate: unclick Auto box, value 6.0
   ix. Red and Blue values: for now do not adjust

59. Place the **XyloTron** so that it is imaging a neutral density white balancing target (e.g. x-rite Color Checker White Balance).

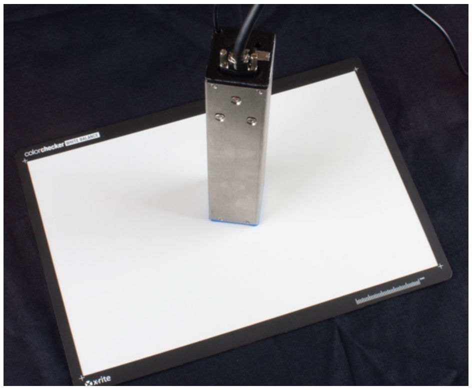

60. Click the histogram button, and click the boxes for Red, Green, and Blue.

61. Adjust the max percent to 10 – you should now have one red curve, one green curve, and one blue curve.

62. Move the histogram window to a part of the screen where you can observe it.

63. Click the “Camera Control Dialog” button to open the control window. Under the “Camera Settings” tab move the red slider and the blue slider until the red and blue curves exactly overlap the green curve. Sometimes the width of the red or blue curve will be slightly narrower or wider than the green curve – in these cases split the distance equally while keeping peak centered with the green peak.

64. This has white-balanced the camera with the LEDs and the lighting position you chose. Record the Red and Blue integer values to the right of the sliders. You will need to convert these (using the provided Excel sheet or Python script) for entry into the camera/lighting/configuration file.

65. Repeat Steps 57-60 for any light source/light position configurations you intend to use – each combination of light source and light position will need its own configuration file.

You have constructed a new XyloTron – your skills are complete.

PART FIVE: DIMENSIONED DRAWINGS OF MACHINED STAINLESS STEEL TUBE

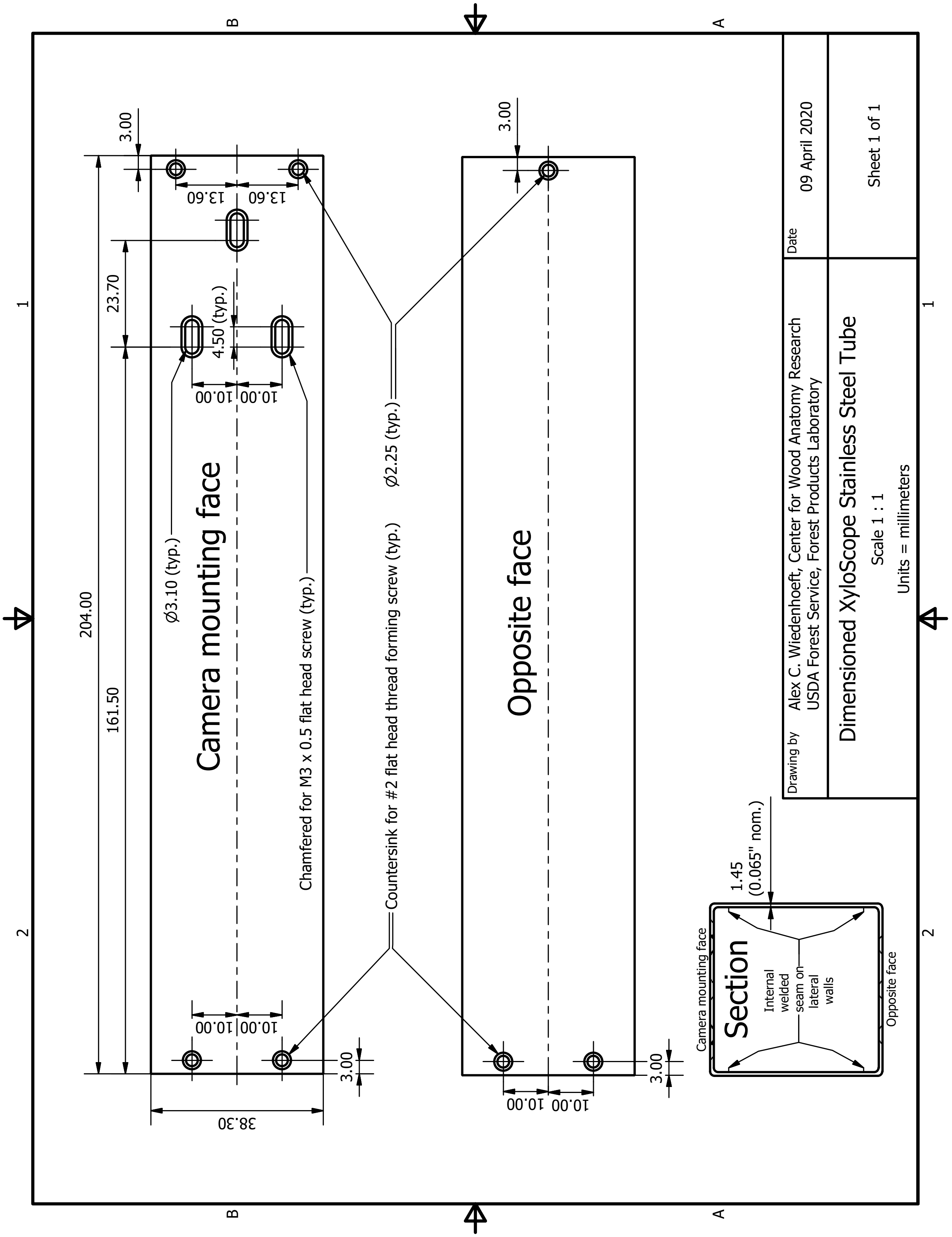

Conventions: 3D printed part names are in **bold case**. Other distinct parts are in *italics*.
Assemblies containing one or more parts of each type are shown in ***italics and bold***.
