# Supplementary material for: The XyloTron: Flexible, Open-Source, Image-Based Macroscopic Field Identification of Wood Products
Source: Front Plant Sci. 2020 Jul 10;11:1015. doi: 10.3389/fpls.2020.01015 (PMC7366520; doi:10.3389/fpls.2020.01015)
Supplement: Supplementary file 4 [file DataSheet_4.pdf]

Supplementary Materials:
XyloTron LED Illumination Circuit

This Supplementary Material describes the custom sample illumination electronics contained within the XyloTron, and uses the part names established in the XyloTron Assembly manual (S1) The XyloTron LED illumination circuit is shown schematically in Figure 1. Renderings of the circuit boards themselves are shown in Figure 2. This document describes how to download and reproduce these custom printed circuit boards. Readers of this document should also refer to the XyloTron assembly guide which contains many helpful pictures and 3D diagrams.

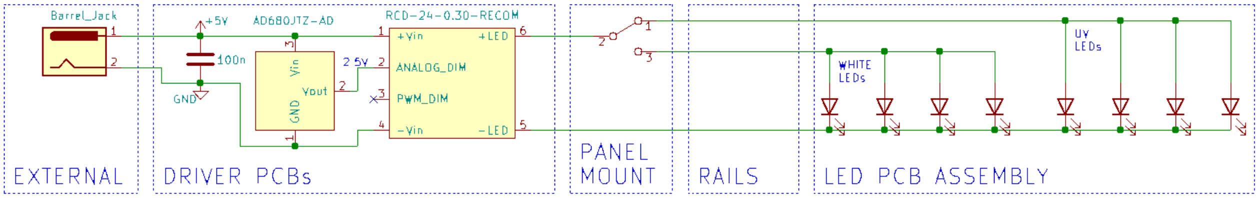

Figure 1. Schematic of LED illumination circuit.

On the “back cap” of the XyloTron there are two external elements related to LED illumination: 1) a barrel jack (Fig. 1, *external*), and 2) a switch (Fig. 1 *panel mount*). The barrel jack (SparkFun TOL-08734) accepts 5 V DC, center positive. This is used to power a constant-current LED driver (RECOM RCD-24-0.30). The LED driver's dimming control pin (labeled ANALOG_DIM, Fig. 1) is held permanently at 2.5V, which corresponds to roughly 100 mA output current.

The externally mounted switch is single-pole-double-throw, sliding (NKK MS13AFG01). This switch directs the output of the LED driver to either the white (Lite-On LTPL-P00DWS57) or UV (SunLED XZVS54S-9A) LEDs. The LEDs are wired in parallel, such that roughly 25 mA is driven through each.

Mechanically, the LED illumination circuitry of the XyloTron is somewhat unusual. There are four unique printed circuit board (PCB) designs, as shown in Figure 2. The boards are rendered to scale relative to each-other, so size comparisons can be made directly. The driver circuitry, built around the RECOM integrated circuit, is split into two separate PCBs due to space considerations. This circuit sits roughly in the middle of the XyloTron, at the thin section of the lens assembly. The 2.1 mm barrel jack used to deliver power is terminated in a 2 pin JST connector, which mounts directly into the power input PCB.

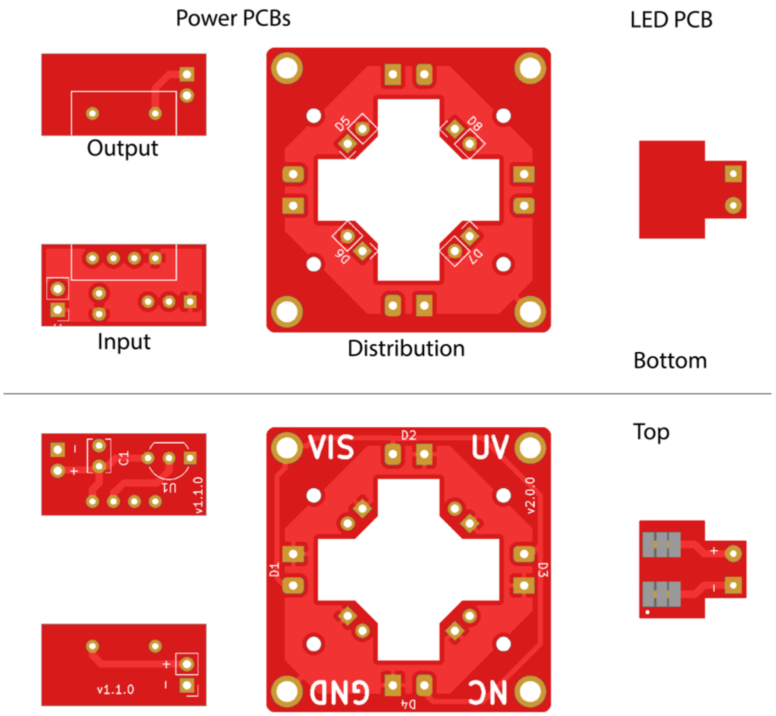

Figure 2. LED illumination printed circuit boards.

Power to the LEDs is delivered directly along the four metal rails that run much the length of the XyloTron internally. The positive output of the power output PCB is routed to the switch (external, panel mounted). The negative output (the return) of the power output PCB is connected directly to one of the four rails within the XyloTron. The two switch outputs are connected to the two other rails. The fourth rail is not used electrically. The large square power distribution PXCB which appears in the center column of Figure 2 has press-fit receptacle in each corner (Mill-Max 0648-0-15-15-23-27-10-0). The four rails go through these receptacles, and good electrical contact is made by the small flex springs inside the contacts. In this way, the square PCB can slide along the length of the rails while receiving power and without worrying about wire bunching or strain.

UV LEDs are soldered onto the bottom of the power distribution board, where the component references D5-D8 appear in the silk screen. Although the LEDs are surface mount (0805 package), through hole pads are used to make hand-soldering easier. The white light LEDs are mounted to small LED PCBs (Figure 2 right-hand side) which slot into the power distribution board at an angle. Small pieces of wire can be soldered between the power distribution PCB and LED PCB boards to make a connection. This assembly is held in place within the 3D-printed plastic LED holder, making a solid unit which slides easily along the powered rails. The XyloTron assembly guide contains pictures of this 3D assembly and bridging wires.

Gerber files, KiCAD source files, and schematic PDFs of each of these four unique PCBs can be found as part of this supplementary information. We have had success with the following for interacting with these files:

- https://kicad-pcb.org – software used to create these PCBs
- https://tracespace.io/view/ - visualize the contents of gerber files
- https://www.pcbway.com/ - order custom PCBs
