# Supplementary material for: The XyloTron: Flexible, Open-Source, Image-Based Macroscopic Field Identification of Wood Products
Source: Front Plant Sci. 2020 Jul 10;11:1015. doi: 10.3389/fpls.2020.01015 (PMC7366520; doi:10.3389/fpls.2020.01015)
Supplement: Supplementary file 5 [file DataSheet_5.pdf]

Supplementary information: XyloTron software applications

Minimum computing requirements:

The XyloTron requires a standard laptop/desktop for image data collection and for deploying the trained models. Since the computer is used only for prediction, no specialized hardware such as GPUs are necessary. In our work a laptop with 8GB RAM has been sufficient.

Three software applications are distributed with the XyloTron:

1. XyloPeek: This application enables quick and easy exploration of the imaging capabilities of the XyloTron.
2. XyloRef: This application was developed for large scale image dataset collection in a xylarium. The main features of the application are automatic image tagging with specimen data from xylarium specimen data spreadsheets (in a standard format), simultaneous imaging and spreadsheet updating for newly added specimens and saving the data in a standardized directory structure. These features minimize the amount of data entry (and thereby metadata errors) and allows the user to concentrate on capturing the most informative images.
3. XyloInf: This software application is used to deploy the trained identification models for testing.

All three software applications provide a live feed, the capability to capture and save images, and the automatic handling of camera parameters based on the substrate and the illumination configuration. The software applications were developed using Python and PyQt. The models were trained and deployed in PyTorch.

Main installation steps:

1. Install Python 3 and PyQt (Anaconda recommended).
2. Install OpenCV.
3. Install PyTorch (version 1.1 and above).
4. Install Spinnaker SDK (downloadable from FLIR)
5. Install PySpin (the Python bindings for Spinnaker, downloadable from FLIR)
6. Copy the xylotron source code to home directory.

Detailed instructions for installation and software usage will be available at https://github.com/fpl-xylotron.
