# Supplementary material for: The XyloTron: Flexible, Open-Source, Image-Based Macroscopic Field Identification of Wood Products
Source: Front Plant Sci. 2020 Jul 10;11:1015. doi: 10.3389/fpls.2020.01015 (PMC7366520; doi:10.3389/fpls.2020.01015)
Supplement: Supplementary file 6 [file DataSheet_6.pdf]

Data sets and machine learning

Prabu Ravindran, Blaise J. Thompson,
Richard K. Soares and Alex C. Wiedenhoeft

1 Data sets

1.1 Wood image data set

Details of the wood image data set - selected species, class labels after species grouping, presence of surface fluorescence and number of images per class - are summarized in Table 1.

1.2 Charcoal image data set

Table 2 provides details of the 6 class charcoal image data set collected for training the charcoal identification model.

2 Machine learning

2.1 Architecture

The convolution neural network (CNN) architecture shown in Figure 2 in the paper was used for the wood and charcoal identification models. The only difference between the two models is the length of the final prediction vector - for wood identification the prediction of the CNN is a vector of length 12 while it is a vector of length 6 for charcoal identification (see Table 3).

2.2 Training and hyperparameters

The models for wood and charcoal identification were trained using a two stage transfer learning methodology [2]. In the first stage, the weights of the

pre-trained backbone were frozen and the (randomly initialized) weights of the custom head were learned. The weights in the entire network were fine tuned in the second stage. Random image patches of size 2048×768 pixels were resized to 512×192 pixels and input to the CNN in mini-batches of size 16. The Adam optimizer [3] was used for both the stages with simultaneous annealing of the learning rate and momentum in two phases [6, 2]. In the first phase the learning rate was increased from α_{min} to α_{max} while the momentum was decreased from β_{max} and β_{min} while in the second phase the learning rate was decreased and the momentum was increased between the same limits as in phase one. Cosine annealing was used for both phases. α_{max} was estimated using the learning rate estimation methodology in [6]. The two stage transfer learning process described here was also used in [4, 5] for wood identification. The hyperparameters are presented in Table 4.

2.3 Data splits

The wood and charcoal data sets were split into 5 folds with class level stratification. The splits were performed in such a way that each specimen contributed images to exactly one of the folds. This constraint allowed the models to be trained and tested on mutually exclusive specimens. In the case of wood identification, this ensures model training and testing was done with *specimens from different trees*. The charcoal specimens were obtained from specimens submitted for forensic verification of commercially available charcoal in Europe. In this case, it was known that the specimens were from geographically distributed locations and multiple vendors, but *tracing each charcoal specimen to the source tree was not possible*.

2.4 Evaluation

Class predictions for the test fold images were obtained using the trained models. The class prediction for a specimen in the test fold was obtained as the majority label of the predictions on its images. Specimen level accuracies and confusion matrices are presented and discussed in the main paper and in this supplement.

Weights of the custom CNN head were randomly initialized (He normal initialization [1]) from 5 different seeds and for each seed five fold cross validation was carried out using the splits and training procedure above. From confusion matrix $C_{s,f}$, for seed s and fold f, an accumulated confusion matrix

C_s for each seed s was obtained as:

$$C_s = \sum_{f=1}^{5} C_{s,f}, \;\; s \in \{1, 2, 3, 4, 5\}.$$

Example confusion matrices for the wood and charcoal identification models (corresponding to one of the seeds) were presented in the main text. The accumulated confusion matrices from the four other seeds for the wood and charcoal models are presented in Figures 1 and 2 respectively. The average prediction accuracies of the wood and charcoal identification models across the 5 seeds are shown in Table 5.

References

[1] Kaiming He, Xiangyu Zhang, Shaoqing Ren, and Jian Sun. Delving deep into rectifiers: Surpassing human-level performance on ImageNet classification. In *Proceedings of the IEEE International Conference on Computer Vision*, pages 1026–1034, 2015.

[2] Jeremy Howard et al. fastai. `https://github.com/fastai/fastai`, 2018.

[3] Diederik P. Kingma and Jimmy Ba. Adam: A method for stochastic optimization. *CoRR*, 2014.

[4] Prabu Ravindran, Emmanuel Ebanyenle, Alberta Asi Ebeheakey, Kofi Bonsu Abban, Ophilious Lambog, Richard Soares, Adriana Costa, and Alex C. Wiedenhoeft. Image based identification of Ghanaian timbers using the XyloTron: Opportunities, risks and challenges. In *NeurIPS Workshop on Machine Learning for the Developing World (ML4D): Challenges and Risks*, 2019.

[5] Prabu Ravindran and Alex C. Wiedenhoeft. Comparison of two forensic wood identification technologies for ten meliaceae woods: computer vision vs. mass spectrometry. *Wood Science and Technology*, (accepted), April 2020.

[6] Leslie N. Smith. A disciplined approach to neural network hyper-parameters: Part 1 - learning rate, batch size, momentum, and weight decay. *CoRR*, abs/1803.09820, 2018.

Label	Species	Image Count
Albizia	*Albizia adianthifolia*<br>*Albizia antunesiana*<br>*Albizia coriaria*<br>*Albizia grandibracteata*<br>*Albizia gummifera*<br>*Albizia lebbeck*	300
Detarium	*Detarium macrocarpum*<br>*Detarium microcarpum*<br>*Detarium senegalense*<br>*Detarium sp*	197
Dialium	*Dialium aubrevillei*<br>*Dialium bipindense*<br>*Dialium dinklagei*<br>*Dialium platysepalum*	49
Hymenaea	*Hymenaea courbaril*<br>*Hymenaea oblongifolia*	300
Inga	*Inga acrocephala*<br>*Inga alba*<br>*Inga bracteosa*<br>*Inga jenmani*	180
Morus	*Morus alba*<br>*Morus australis*<br>*Morus rubra*	300
Nauclea	*Nauclea diderrichii*<br>*Nauclea orientalis*	300
Robinia	*Robinia neo-mexicana*<br>*Robinia pseudoacacia*	300
Swietenia	*Swietenia macrophylla*	300
Tectona	*Tectona grandis*	300
U.americana	*Ulmus americana*	300
U.rubra	*Ulmus rubra*	300

Table 1: Wood data set details. 3126 images were collected from 470 wood specimens. The 31 species were divided into 12 classes. Classes that exhibit surface fluorescence are highlighted.

Label	Image Count
Acer	300
Betula	300
Carpinus	193
Fagus	167
Fraxinus	230
Quercus	122

Table 2: Details of the 6 class charcoal dataset. 1312 images were collected from 150 charcoal specimens.

Model	N_c
Wood	12
Charcoal	6

Table 3: The length of the prediction vector (represented as N_c in Figure X in the paper) for the two models presented.

Hyperparameter	Value
Patch size	512×192 (pixels)
Minibatch size	16
Number of epochs (stage 1)	10
Number of epochs (stage 2)	8
α_{max} (stage 1)	$2e^{-2}$
α_{max} (stage 2)	$1e^{-5}$
α_{min} (stages 1, 2)	$\alpha_{\text{max}}/10$
β_{max}	0.95
β_{min}	0.85

Table 4: The hyperparameters used for training the wood and charcoal models.

Model	Accuracy (%)
Wood	96.4 ± 0.7
Charcoal	97.5 ± 0.9

Table 5: Prediction accuracies averaged over the five weight initializations.

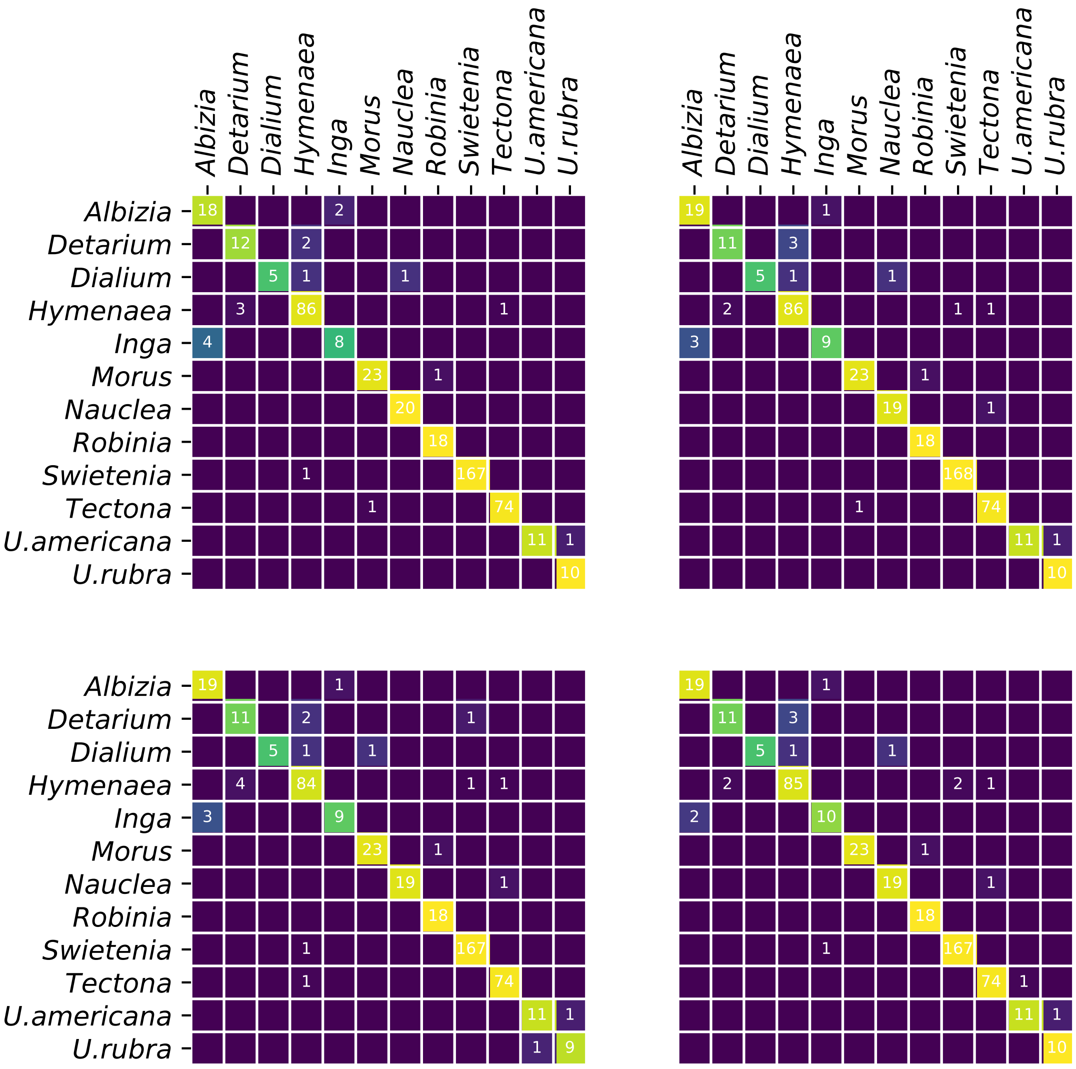

Figure 1: Confusion matrices for wood identification models. Cell colors are coded by accuracy percentages. Annotations are provided for cells with non-zero specimen counts.

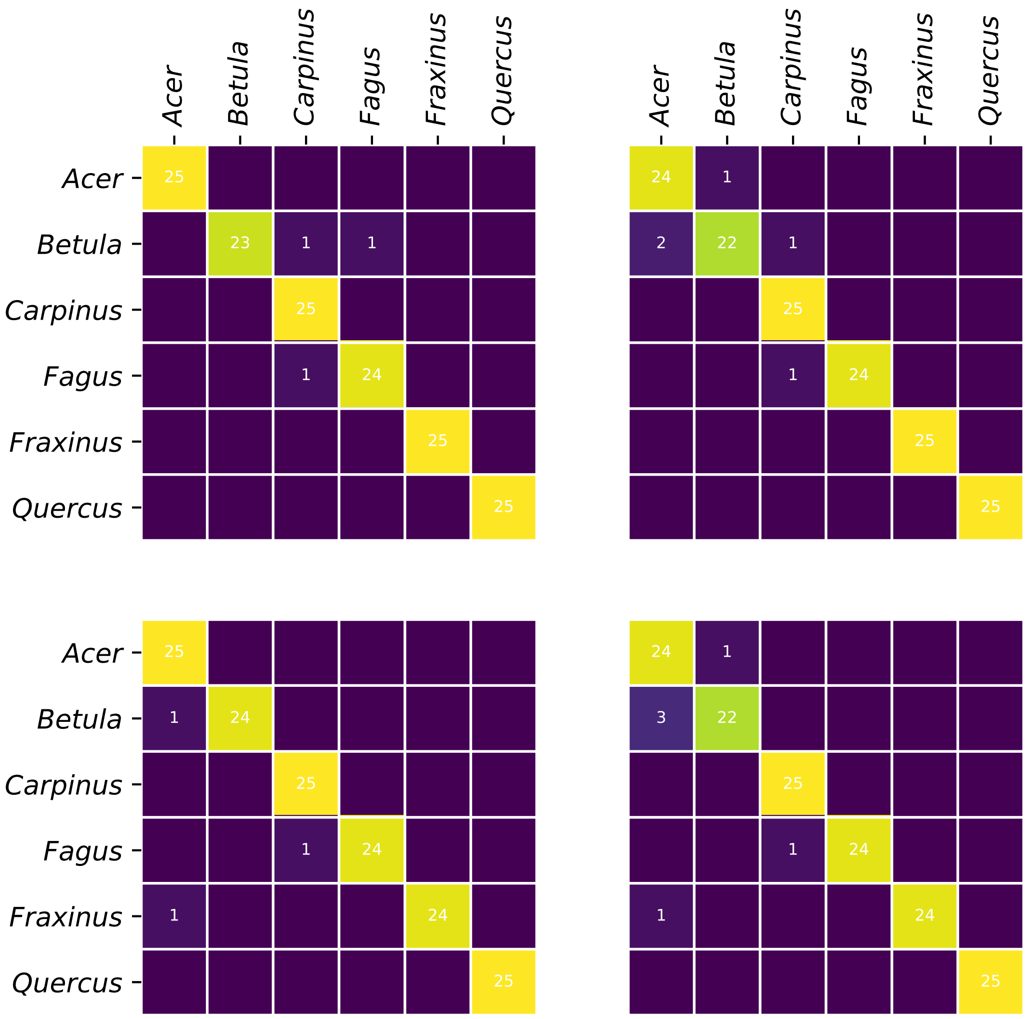

Figure 2: Confusion matrices for charcoal identification models. Cell colors are coded by accuracy percentages. Annotations are provided for cells with non-zero specimen counts.
