# Supplementary material for: The XyloTron: Flexible, Open-Source, Image-Based Macroscopic Field Identification of Wood Products
Source: Front Plant Sci. 2020 Jul 10;11:1015. doi: 10.3389/fpls.2020.01015 (PMC7366520; doi:10.3389/fpls.2020.01015)

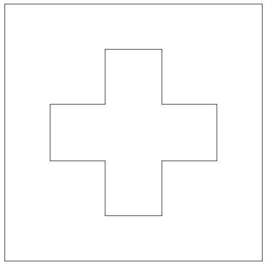

Supplement: Supplementary file 2 [file DataSheet_2.zip › Electronics_files/distribution-kicad.zip!/distribution/distribution-pcb.pdf]
