# Supplementary material for: The XyloTron: Flexible, Open-Source, Image-Based Macroscopic Field Identification of Wood Products
Source: Front Plant Sci. 2020 Jul 10;11:1015. doi: 10.3389/fpls.2020.01015 (PMC7366520; doi:10.3389/fpls.2020.01015)
Supplement: Supplementary file 2 [file DataSheet_2.zip › Electronics_files/LED-kicad.zip!/daughter/LED.pdf]

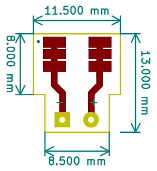

bthompson@chem.wisc.edu
Blaise Thompson
Department of Chemistry
UW–Madison

Sheet:
File: LED.kicad_pcb

Title: LED PCB

Size: USLetter	Date: 2019-09-13	Rev: C
KiCad E.D.A. kicad 5.1.4+dfsg1-1		Id: 1/1
