# Supplementary material for: The XyloTron: Flexible, Open-Source, Image-Based Macroscopic Field Identification of Wood Products
Source: Front Plant Sci. 2020 Jul 10;11:1015. doi: 10.3389/fpls.2020.01015 (PMC7366520; doi:10.3389/fpls.2020.01015)
Supplement: Supplementary file 2 [file DataSheet_2.zip › Electronics_files/LED-kicad.zip!/daughter/front.pdf]

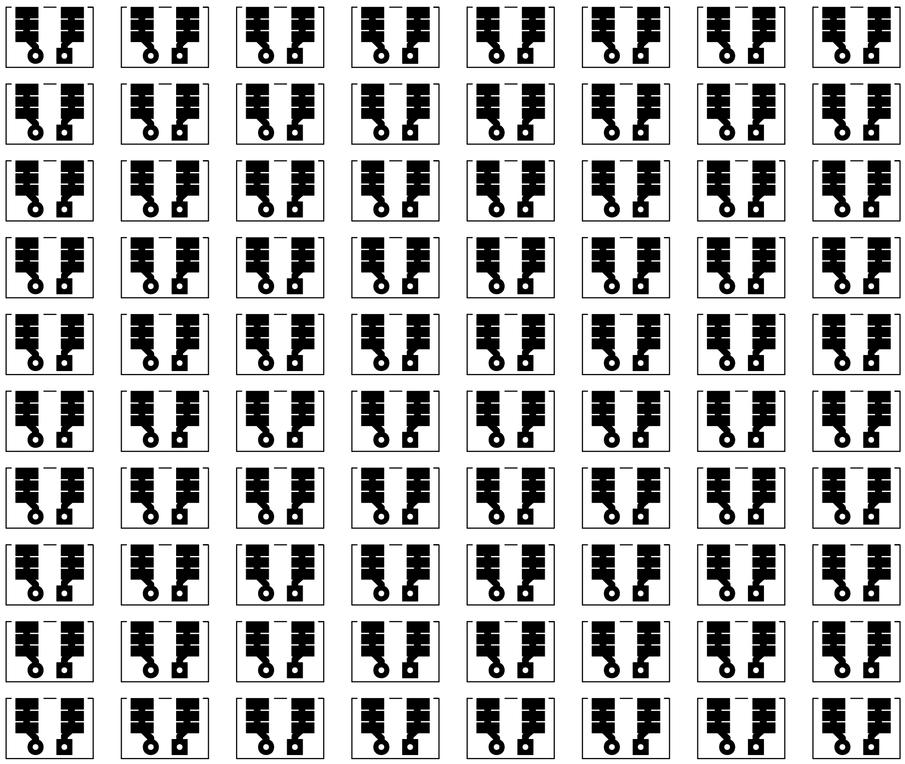

**Title: LED PCB**

Date: 2018-12-12

Rev: B

Desiged by Blaise Thompson, UW-Madison, Department of Chemistry

Size: USLetter
