# Supplementary material for: The XyloTron: Flexible, Open-Source, Image-Based Macroscopic Field Identification of Wood Products
Source: Front Plant Sci. 2020 Jul 10;11:1015. doi: 10.3389/fpls.2020.01015 (PMC7366520; doi:10.3389/fpls.2020.01015)
Supplement: Supplementary file 2 [file DataSheet_2.zip › Electronics_files/supply-kicad.zip!/supply_PCB/supply.pdf]

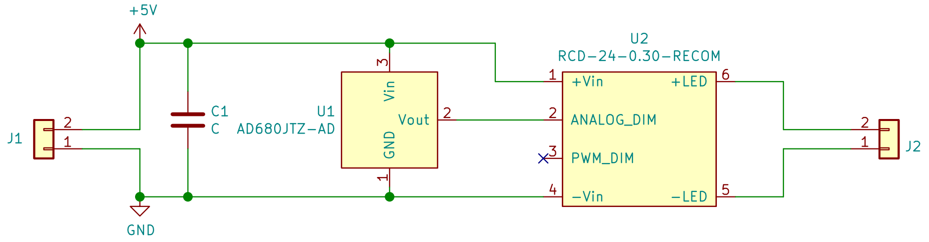

bthompson@chem.wisc.edu
Blaise Thompson
University of Wisconsin-Madison
Wiedenhoeft Group

Sheet: /
File: supply.sch

Title: XyloScope LED Driver

Size: USLetter	Date: 2020-02-05	**Rev: 1.1.0**
KiCad E.D.A. kicad 5.1.5+dfsg1-2		Id: 1/1
